# Patient Out-of-Pocket Costs for Biologic Drugs After Biosimilar Competition

**DOI:** 10.1001/jamahealthforum.2023.5429

**Published:** 2024-03-29

**Authors:** Kimberly Feng, Massimiliano Russo, Luca Maini, Aaron S. Kesselheim, Benjamin N. Rome

**Affiliations:** 1 Program on Regulation, Therapeutics, and Law, Division of Pharmacoepidemiology and Pharmacoeconomics, Department of Medicine, Brigham and Women’s Hospital, Boston, Massachusetts; 2 Harvard Medical School, Boston, Massachusetts; 3 Department of Medicine, Beth Israel Deaconess Medical Center, Boston, Massachusetts; 4 Department of Health Care Policy, Harvard Medical School, Boston, Massachusetts

## Abstract

**Importance:**

Biologic drugs account for a growing share of US pharmaceutical spending. Competition from follow-on biosimilar products (subsequent versions that have no clinically meaningful differences from the original biologic) has led to modest reductions in US health care spending, but these savings may not translate to lower out-of-pocket (OOP) costs for patients.

**Objective:**

To investigate whether biosimilar competition is associated with lower OOP spending for patients using biologics.

**Design, Setting, and Participants:**

This cohort study used a national commercial claims database (Optum Clinformatics Data Mart) to identify outpatient claims for 1 of 7 clinician-administered biologics (filgrastim, infliximab, pegfilgrastim, epoetin alfa, bevacizumab, rituximab, and trastuzumab) from January 2009 through March 2022. Claims by commercially insured patients younger than 65 years were included.

**Exposure:**

Year relative to first biosimilar availability and use of original or biosimilar version.

**Main Outcomes and Measures:**

Patients’ annual OOP spending on biologics for each calendar year was determined, and OOP spending per claim between reference biologic and biosimilar versions was compared. Two-part regression models assessed for differences in OOP spending, adjusting for patient and clinical characteristics (age, sex, US Census region, health plan type, diagnosis, and place of service) and year relative to initial biosimilar entry.

**Results:**

Over 1.7 million claims from 190 364 individuals (median [IQR] age, 53 [42-59] years; 58.3% females) who used at least 1 of the 7 biologics between 2009 and 2022 were included in the analysis. Over 251 566 patient-years of observation, annual OOP costs increased before and after biosimilar availability. Two years after the start of biosimilar competition, the adjusted odds ratio of nonzero annual OOP spending was 1.08 (95% CI, 1.04-1.12; *P* < .001) and average nonzero annual spending was 12% higher (95% CI, 10%-14%; *P* < .001) compared with the year before biosimilar competition. After biosimilars became available, claims for biosimilars were more likely than reference biologics to have nonzero OOP costs (adjusted odds ratio, 1.13 [95% CI, 1.11-1.16]; *P* < .001) but had 8% lower mean nonzero OOP costs (adjusted mean ratio, 0.92 [95% CI, 0.90-0.93; *P* < .001). Findings varied by drug.

**Conclusions and Relevance:**

Findings of this cohort study suggest that biosimilar competition was not consistently associated with lower OOP costs for commercially insured outpatients, highlighting the need for targeted policy interventions to ensure that the savings generated from biosimilar competition translate into increased affordability for patients who need biologics.

## Key Points

**Question:**

Among US commercially insured patients using biologic drugs, is competition by biosimilars associated with lower out-of-pocket (OOP) spending?

**Findings:**

In this cohort study of 190 364 outpatients with 1.7 million claims for 7 biologics between 2009 and 2022, annual OOP spending did not decrease after the start of biosimilar competition, and OOP costs were similar for biosimilars and their reference biologics.

**Meaning:**

Findings of this study suggest that the introduction of biosimilar competition did not systematically lower patient OOP spending on biologics, highlighting the need for targeted policy interventions to ensure that savings from biosimilar competition improves affordability for patients.

## Teaser

This cohort study examines whether the availability of biosimilar drugs is associated with lower out-of-pocket costs for biologic medications among commercially insured patients in the US.

## Introduction

Patients and payers in the US spend more on prescription drugs than any other country in the world, with spending exceeding $500 billion in 2021.^1,2,3^ These expenses are driven disproportionately by high-cost biologics, which are complex drugs produced in living systems and often require special physician administration (eg, intravenously).^4,5,6^ To address the growing costs of biologics, policymakers introduced an abbreviated regulatory pathway for biosimilars, which are subsequent versions of biologic products with no clinically meaningful differences from an existing US Food and Drug Administration (FDA)–approved reference product.^7^ Similar to generic versions of small-molecule drugs, biosimilars can enter the market after the expiration of market-exclusivity protection on the original product, and provide much-needed price competition that can lead to reduced spending.

Prices for biosimilars are typically 15% to 35% lower than their respective brand-name reference biologic^8,9^ and can prompt the brand name manufacturers to lower prices or offer discounts.^10,11^ Altogether, biosimilars have produced nearly $13 billion in savings since 2015^9^ and are projected to save between $38 and $124 billion from 2021 to 2025.^8,12^ Although these system-wide savings lower the cost of health care for all consumers, it is less clear whether biosimilar competition lowers the costs borne individually by patients using biologics.

Costs of many biologics are reimbursed under medical insurance benefits as opposed to pharmaceutical benefits. Patient cost-sharing for medical services is determined by specific benefit design features; these costs often vary throughout the year depending on when patients meet deductibles and out-of-pocket (OOP) maximums. In addition, reimbursement rates negotiated between insurers and hospitals or clinics vary, and frequently can exceed the sales price of the medication.^13^ As a result, it is plausible that savings generated from biosimilar competition could lower premiums for all patients without markedly reducing OOP costs for the patients who take these medications.

Whether biosimilar competition leads to lower OOP costs has important implications for affordability and access to these medications, as high OOP costs are associated with lower medication initiation and adherence, increased financial stress, and worse clinical outcomes.^14,15,16^ In this study, we assessed OOP costs among commercially insured patients using 7 clinician-administered biologics with biosimilars available in the US as of January 2021. We investigated whether annual OOP costs decreased after the introduction of biosimilar competition, and whether OOP costs were lower for patients using biosimilars when compared with the brand name biologic.

## Methods

### Data Source

We used data from Optum Clinformatics Data Mart, a large national administrative health claims database of commercially insured individuals. The Massachusetts General Brigham Institutional Review Board approved the study and waived the informed consent requirement because only deidentified claims were used. Results of this cohort study were reported in accordance with Strengthening the Reporting of Observational Studies in Epidemiology (STROBE) guideline.

We focused on outpatient medical claims for biologics with biosimilar versions available in the US prior to January 2021 (Table 1). Eligible drugs were identified using the FDA Biosimilar Product Information list.^17^ As of January 2021, 11 biologics had FDA-approved biosimilar versions; we excluded 3 drugs whose approved biosimilars were not yet marketed in the US due to ongoing patent litigation (adalimumab, etanercept, and ranibizumab).^18^ We also excluded insulin glargine, the only pharmacy-administered drug with biosimilar competition, because its cost-sharing and benefit design differ from those of clinician-administered drugs. One drug, filgrastim, had a follow-on product (tbo-filgrastim) approved in 2013 before the abbreviated biosimilar pathway was available; however, we included this follow-on drug as a biosimilar.

**Table 1.** Characteristics of Biologics Included in the Study

Biologic drug	Primary areas of therapeutic use	First biosimilar competition	No. of biosimilars^a^	No. of claims in year before biosimilar competition	No. of claims for biosimilar (% of total claims)^b^
Filgrastim	Hematologic, oncologic	November 2013^c^	3	16 591	51 588 (50)
Infliximab	Gastrointestinal, rheumatologic	November 2016	3	43 395	32 165 (13)
Pegfilgrastim	Hematologic, oncologic	July 2018	3	21 172	6451 (9)
Epoetin alfa	Renal, hematologic, oncologic	November 2018	1	5775	6853 (42)
Bevacizumab	Oncologic, ophthalmologic	July 2019	2	30 228	19 123 (25)
Rituximab	Oncologic, rheumatologic	November 2019	3	11 349	9423 (42)
Trastuzumab	Oncologic	July 2019	5	19 805	23 830 (59)

^a^ Available in the US market as of December 31, 2021.

^b^ Among all claims for the biologic from the date of first biosimilar competition entry into the market through March 31, 2022.

^c^ The first filgrastim follow-on product, tbo-filgrastim, was approved before the abbreviated biosimilar approval pathway was available. Because it was made by a competing manufacturer and approved for similar use as the brand-name medication, tbo-filgrastim was included in the study.

### Patient Cohort

We investigated claims from January 1, 2009 (4 years before the first biosimilar was marketed), through March 31, 2022 (end of available data). For each drug, we identified relevant claims using Healthcare Common Procedure Coding System codes for outpatient medical services (eTable 1 in Supplement 1). We included claims only for adults younger than 65 years with commercial insurance plans, because Medicare Advantage plans may have different reimbursement policies and cost-sharing requirements. We also excluded inpatient claims, because itemized costs for inpatient medications are not well recorded in claims data and may differ from outpatient costs owing to differences in insurance benefit design.

### Outcome

The primary outcome was patient OOP cost, including deductible, copayment, and coinsurance. When multiple claims were filed for the same patient on the same service date for the same drug (ie, a claim adjustment), we summed OOP costs across claims. We removed fewer than 1% of claims with negative OOP cost variables after adjustment, as these likely represented claims with missing or inaccurate data. Each claim included a database-encrypted identifier linking claims of individual patients over time, allowing us to calculate total OOP spending per patient during each calendar year.

We also measured covariates, including age, sex, US Census region, health plan type, place of service, and primary diagnosis code, associated with the claim. Health plan type was stratified into high-deductible vs non–high-deductible plans; high-deductible plans included those coupled with a health savings account^19^ as well as consumer-driven health plans, which are preferred provider organization plans coupled with health reimbursement arrangements and typically also have high deductibles. For diagnoses, we included *International Classification of Diseases, Ninth Revision* (through September 2015) and *International Statistical Classification of Diseases and Related Health Problems, Tenth Revision* (October 2015 and later) codes consolidated into 7 clinical categories based on the major FDA-approved indications and other uses for each drug: hematologic, oncologic, rheumatologic, renal, gastrointestinal, ophthalmologic, and neurologic (Table 1 and eTable 2 in Supplement 1).

### Statistical Analysis

We performed 2 analyses to measure the association between biosimilar competition and OOP costs. First, we assessed trends in annual OOP costs for patients using biologics before and after biosimilar competition began. In the first analysis, we studied each biologic for up to 4 calendar years before and after the year of first biosimilar availability. Second, we compared patient OOP spending per claim between reference biologics and biosimilar versions to investigate whether biosimilars were less costly for patients to use. For the second analysis, we included claims from the first full calendar year after availability of a biosimilar version through the end of available data.

In both analyses, the OOP cost data were right-skewed and included a large proportion of zeros (ie, the medication cost was fully covered by insurance). To address this, we used a 2-part modeling approach that involved first modeling the odds of nonzero OOP costs, then modeling mean OOP costs in the subset of patients with nonzero OOP spending.^20^ Analyses were performed in R, version 4.2.0 (R Project for Statistical Computing).

#### Trends in Annual OOP Costs

In the first analysis, we summed annual OOP spending on biologics (including the reference biologic and any biosimilars) for patients with at least 1 claim for such an agent during the calendar year. We centered analyses around the year during which a biosimilar version initially entered the market (year 0).

We used logistic regression to estimate the odds ratio (OR) of nonzero annual OOP spending each year relative to the year immediately before market entry of the first biosimilar (year −1). Next, among patients with nonzero annual OOP spending, we used generalized linear regression with a γ distribution and log link to determine the ratio of mean nonzero annual OOP costs each year compared with year −1. In both models, we adjusted for patient and clinical characteristics (age, sex, US Census region, health plan type, primary clinical diagnosis category, place of service [outpatient hospital, office, or other, including home, hospice, skilled nursing facilities, and dialysis centers]), and year relative to initial biosimilar market entry. The SEs were clustered by patient to account for patients being included in multiple years.

We modeled each drug separately and then estimated the average trend for all 7 drugs using a random-effects 2-part model. For this model, we used the same 2-part approach and the same fixed-effect covariates as described previously, but included random effects for the drugs in both parts of the model to account for heterogeneity among biologics.

#### OOP Costs for Biosimilars vs Reference Biologics

In the second analysis, we used logistic regression to estimate the OR of nonzero OOP costs among claims for biosimilars compared with claims for each of the original 7 biologics. Among those claims with nonzero OOP costs, we used a generalized linear model with γ distribution and log link to compare the ratio of the mean OOP costs between the 2 groups. We adjusted for the same patient and clinical characteristics as described previously as well as calendar month and year. Adjusting for month was important because average OOP costs were highest early in the year and decreased as patients met their insurance plan deductibles or OOP maximums. We performed post hoc sensitivity analyses to assess the robustness of the findings by repeating the models without including age and sex as covariates.

## Results

The study included 7 biologics that faced new biosimilar competition between November 2013 (filgrastim) and July 2019 (trastuzumab). These drugs were approved to treat cancers, hematologic disorders, and inflammatory and autoimmune diseases (Table 1). As of January 2021, epoetin alfa had only 1 biosimilar; the other 6 biologics had at least 2 biosimilars, with a maximum of 5 for trastuzumab. We identified a total of 1.7 million claims from 190 364 individuals (median [IQR] age, 53 [42-59] years; 146 579 females [58.3%] and 104 987 males [41.7%]) who used at least 1 of the 7 biologics from January 1, 2009, through March 31, 2022. Subsets of these data were used for the 2 analyses.

### Trends in Annual OOP Costs

For the first analysis, we included a total of 1.3 million claims from 145 389 individuals that occurred during the 4 years before and after biosimilar competition began for each drug. We included at least 2 years of data after biosimilar availability for each drug; only filgrastim and infliximab had 4 full years of postcompetition data. The analytic cohort included 251 566 patient-years. Overall, 66% of patient-years were contributed by patients aged 45 to 64 years, 58% of patients were female, 26% were enrolled in high-deductible health plans, and 60% received the drug primarily in an office setting (Table 2). Infliximab was the most common biologic used, comprising 31% of patient-years, and epoetin alfa was the least common, making up only 3% of patient-years.

**Table 2.** Baseline Characteristics of Patients Using Biologics Included in the Annualized Spending Model

Characteristic	Patient-years, No. (%) (N = 251 566)
Age, y	
0-17	8526 (3.4)
18-44	76 317 (30.3)
45-64	166 723 (66.3)
Sex	
Female	146 579 (58.3)
Male	104 987 (41.7)
US Census region	
Northeast	22 969 (9.1)
Midwest	70 421 (28.0)
South	111 641 (44.4)
West	46 535 (18.5)
Primary site of service	
Office	151 304 (60.1)
Outpatient hospital	88 356 (35.1)
Other^a^	11 906 (4.7)
High-deductible health plan	
No	186 579 (74.2)
Yes	64 987 (25.8)
Biologic	
Filgrastim	23 638 (9.4)
Infliximab	79 038 (31.4)
Pegfilgrastim	50 110 (19.9)
Epoetin alfa	8912 (3.5)
Bevacizumab	51 875 (20.6)
Rituximab	24 556 (9.8)
Trastuzumab	13 437 (5.3)

^a^ Other primary site of service includes home, hospice, skilled nursing facilities, and dialysis centers.

**Figure 1.**
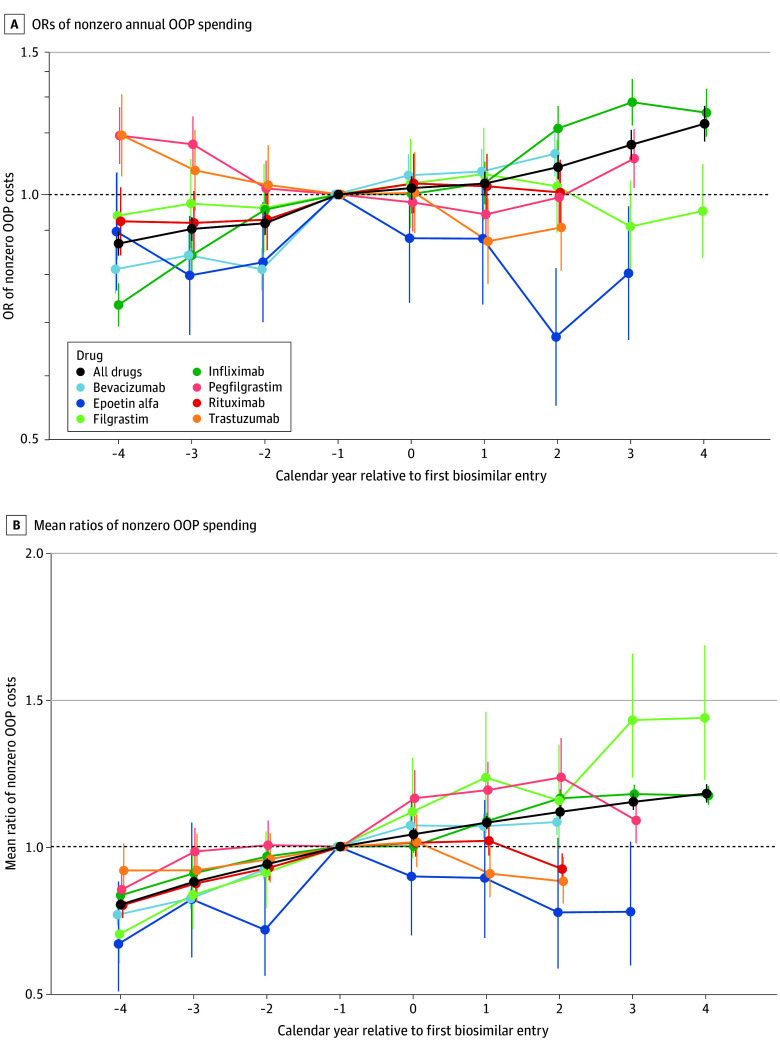
Trends in Annual Out-of-Pocket (OOP) Spending for Biologic Drugs, Before and After Biosimilar Availability All OOP costs are compared with costs in the year before biosimilar competition entered the market (year −1). The average for all drugs was determined by a mixed model that included random effects estimates for all 7 drugs examined. Odds ratios (ORs) greater than 1 indicate more patients paying OOP annually, ORs less than 1 indicate fewer patients paying OOP annually. Mean ratios greater than 1 indicate higher mean OOP costs, mean ratios less than 1 indicate lower OOP costs. Whiskers represent 95% CIs.

For nearly half of the patient-years (122 784; 49%), patients had nonzero annual OOP spending. Averaging all 7 drugs, there was a trend toward a greater share of patients with nonzero OOP costs and higher mean nonzero annual OOP costs both before and after biosimilars entered the market (Figure 1). Compared with the year before biosimilar availability, in the second year after competition entered the market, the adjusted OR of nonzero annual OOP spending was 1.08 (95% CI, 1.04-1.12; *P* < .001) (Figure 1A) and mean nonzero annual spending was 12% higher (adjusted mean ratio [AMR], 1.12 [95% CI, 1.10-1.14]; *P* < .001) (Figure 1B).

Trends in OOP spending varied by drug (Figure 1; full model results in eTable 3 in Supplement 1). The 2 biologics with the most recent biosimilar competition (rituximab and trastuzumab) were the only drugs to show significantly lower mean nonzero annual OOP spending 2 years after biosimilar entry (rituximab: AMR, 0.92 [95% CI, 0.87-0.98]; *P* = .005 and trastuzumab: AMR, 0.88 [95% CI, 0.81-0.96]; *P* = .006); the other 5 drugs had either higher or nonsignificant changes in mean nonzero OOP spending (Figure 1B).

### OOP Costs for Biosimilars vs Reference Biologics

In the second analysis, we included 586 493 claims for 81 197 individuals who used any of the 7 biologics after a biosimilar became available. In all, 149 433 claims (25%) were for a biosimilar version, representing 28 352 individuals (35%). The percentage of claims for biosimilars ranged from 9% of pegfilgrastim claims to 59% of trastuzumab claims (Table 1).

**Figure 2.**
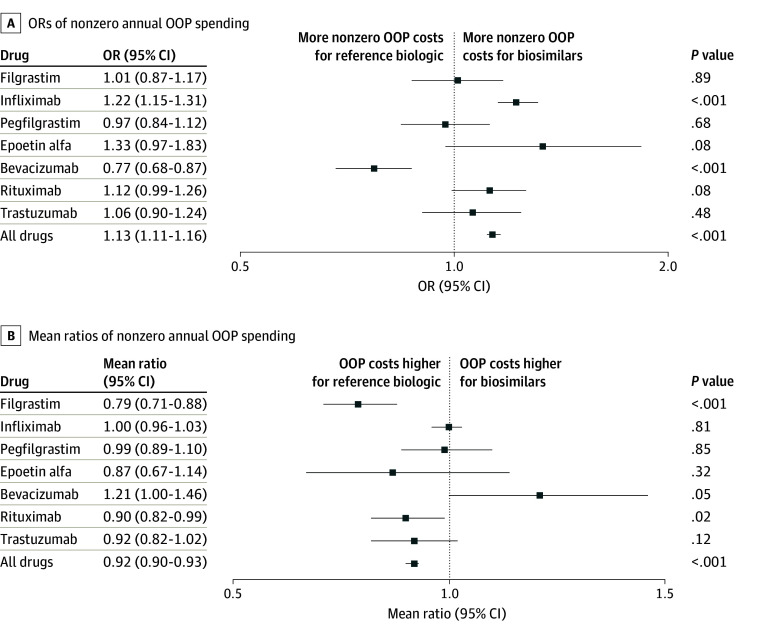
Differences in Out-of-Pocket (OOP) Spending Between Claims for Biosimilars and Reference Biologics Odds ratios (ORs) less than 1 indicate fewer patients paying OOP costs for biosimilar versions compared with the reference biologic. Mean ratios less than 1 indicate lower OOP costs for the biosimilar versions compared with the reference biologic. The average for all drugs was determined by a mixed model that included random effects estimates for all 7 drugs. Whiskers represent 95% CIs.

Overall, 28% of reference biologic claims and 17% of biosimilar claims had nonzero OOP costs. After adjusting for covariates, biosimilar claims were more likely to have nonzero OOP costs than claims for the reference biologic (adjusted OR [AOR], 1.13 [95% CI, 1.11-1.16]; *P* < .001) (Figure 2A and eTable 4 in Supplement 1). There was substantial variation in nonzero OOP costs among the 7 drugs. For example, claims for biosimilar infliximab were more likely to have nonzero OOP costs than the reference biologic (AOR, 1.22 [95% CI, 1.15-1.31]; *P* < .001), while claims for bevacizumab demonstrated the opposite trend (AOR, 0.77 [95% CI, 0.68-0.87]; *P* < .001).

Among claims with nonzero OOP costs, the mean OOP costs were lower for biosimilars ($707) than reference biologics ($911). In the mixed model, adjusted mean nonzero OOP costs were 8% lower for biosimilars compared with reference biologics (AMR, 0.92 [95% CI, 0.90-0.93]; *P* < .001) (Figure 2B; full model results in eTable 4 in Supplement 1). Again, there was variation by drug: mean nonzero OOP costs were lower for biosimilar filgrastim (AMR, 0.79 [95% CI, 0.71-0.88]; *P* < .001) and rituximab (AMR, 0.90 [95% CI, 0.82-0.99]; *P* = .02), but higher for biosimilar bevacizumab (AMR, 1.21 [95% CI, 1.00-1.46]; *P* = .05) compared with the original versions of these drugs. In sensitivity analyses, model results were unchanged after age and sex were removed as covariates (eTable 5 in Supplement 1).

## Discussion

In the past decade, biosimilar competition has not been systematically associated with lower OOP spending among commercially insured patients using biologics. This study found that annual OOP costs increased or remained stable for most biologics even after biosimilar competition began, and patients who used biosimilars did not pay less OOP than those who used reference biologics. These trends varied widely by drug, further emphasizing that biosimilar competition has not consistently reduced patient OOP costs. Additional regulatory attention is needed to ensure that savings generated by biosimilar competition translate into improved patient affordability and access to biologics.

Previous studies examining the association between biosimilars and OOP costs have yielded mixed results. A study of filgrastim found a decrease in monthly OOP costs after biosimilar entry only for the high-deductible health plan population.^21^ Studies of infliximab found that biosimilar use was associated with either no change^22^ or only modestly decreased OOP costs per claim; however projected annual OOP spending was higher for the biosimilar due to cost-sharing and lack of discounts.^23^ Finally, a study of pegfilgrastim found that OOP costs per cycle were lower for biosimilar users.^24^ These mixed findings align with the variations between drugs that we observed. One promising sign from our data was a signal toward lower OOP costs after entry of newer biosimilars, such as trastuzumab and rituximab, although more data are needed to determine if this trend will continue.^12,25^

There are several potential reasons why biosimilar competition has not consistently led to OOP savings for patients using these drugs. First, patient cost-sharing depends on insurance benefit design; costs vary throughout the calendar year as patients meet deductibles and OOP maximums. This complexity might limit the ability to determine direct associations between drug costs and patient OOP spending, even among patients who pay coinsurance (a percentage of the drug’s cost). Second, patient OOP costs for clinician-administered drugs are based on the amount reimbursed by insurers to hospitals and clinics; for commercially insured patients, these reimbursement rates are frequently much higher than the cost of the drug.^13,26^ By contrast, Medicare reimburses for clinician-administered drugs based on the average sales price (the average discounted price at which manufacturers sell the drug). Thus, our results are not generalizable to Medicare patients, for whom OOP costs may be more closely tied to drug prices.

The savings generated by biosimilars to date are associated with lower health care spending and likely lower premiums, but these savings may be too modest to directly impact OOP costs.^11,25^ This may be in part due to limited competition: 6 of the 7 biologics in our study had 3 or fewer biosimilar versions as of January 2021, and studies of small-molecule drugs have shown that competition by more than 3 generics is associated with meaningful reductions in price.^27,28^ Due to the short time frame of this study, we could not analyze whether the number of biosimilar competitors was associated with lower OOP costs for patients. Billing rules are another factor that might hinder competition for clinician-administered biologics. Reference biologics and biosimilars each have separate billing codes, unlike small-molecule drugs and generics, which share a billing code.^29^ Because of this, lower average sales prices for 1 biosimilar do not directly affect the average sales prices for the reference product or for other biosimilars, thereby limiting direct competition.^30^

While strengthening the biosimilar market and further incentivizing biosimilar uptake are important, it is equally important to ensure that biosimilar competition translates into better affordability and access for patients who rely on these medications. For privately insured patients, state or federal laws could constrain OOP costs for biosimilars; alternatively, legislators could seek to ensure that patient cost-sharing is not tied to reimbursement rates that can exceed drug prices.

### Limitations

This study has several limitations. We lacked data about individuals’ specific insurance benefit designs; as a result, we combined deductibles, copayments, and coinsurance into a single OOP costs variable, which better reflects patients’ experiences but misses differences between forms of cost-sharing and does not capture any changes in premium payments. We also lacked robust unit data to allow for adjustments in cost based on amount of drug, although we have no reason to suspect such differences would confound our results. Additionally, our assessment of OOP costs was based on insurance claims, which do not include manufacturer coupons or patient assistance programs that may offset some or all of these expenses.^31^

Results of the present study are limited to clinician-administered biologics and likely are not generalizable to pharmacy-administered biologics, such as insulins or adalimumab. However, even for pharmacy-administered biologics, competition may not translate to lower OOP costs, as some biosimilar makers have set prices similar to the brand name biologic and offered confidential rebates to negotiate formulary position with insurers and pharmacy benefit managers^32^; rebates like these are typically not reflected as lower patient OOP costs.^33^ Finally, the association between biosimilar competition and OOP costs might change in the future. Numerous biosimilars have been approved since 2021, so future studies will need to examine whether more robust competition and the proliferation of interchangeable biosimilars have augmented savings for patients.

## Conclusions

The findings from this cohort study suggest that for commercially insured patients, biosimilar competition thus far has not been associated with lower patient OOP costs. Policymakers should take additional steps to assure that biosimilar competition makes biologics and biosimilars affordable and accessible to the patients who need them.

## Notes

Supplement 1. **eTable 1.** List of All Biologic Drugs and Biosimilar Formulations Available Prior to January 1, 2021, With Relevant Healthcare Common Procedure Coding Codes Used for Identification of Claims **eTable 2.** *ICD-9* and *ICD-10* Code Prefixes Used to Group Claims by Clinical Category for Analysis Models **eTable 3.** Models of Annual Out-of-Pocket Spending in the 4 Years Before and After Biosimilar Entry **eTable 4.** Models of Out-of-Pocket Spending per Claim in the Period After Biosimilars Were Available **eTable 5.** Sensitivity Analyses: Combined Effects Models Without Age and Sex

Supplement 2.
Data Sharing Statement
