# Supplementary material for: Patient Out-of-Pocket Costs for Biologic Drugs After Biosimilar Competition
Source: JAMA Health Forum. 2024 Mar 29;5(3):e235429. doi: 10.1001/jamahealthforum.2023.5429 (PMC10980968; doi:10.1001/jamahealthforum.2023.5429)
Supplement: Supplement 1. — eTable 1. List of All Biologic Drugs and Biosimilar Formulations Available Prior to January 1, 2021, With Relevant Healthcare Common Procedure Coding Codes Used for Identification of Claims eTable 2. ICD-9 and ICD-10 Code Prefixes Used to Group Claims by Clinical Category for Analysis Models eTable 3. Models of Annual Out-of-Pocket Spending in the 4 Years Before and After Biosimilar Entry eTable 4. Models of Out-of-Pocket Spending per Claim in the Period After Biosimilars Were Available eTable 5. Sensitivity Analyses: Combined Effects Models Without Age and Sex [file jamahealthforum-e235429-s001.pdf]

Supplemental Online Content

Feng K, Russo M, Maini L, Kesselheim AS, Rome BN. Patient out-of-pocket costs for biologic drugs after biosimilar competition. *JAMA Health Forum*. 2024;5(3):e235429. doi:10.1001/jamahealthforum.2023.5429

eTable 1. List of All Biologic Drugs and Biosimilar Formulations Available Prior to January 1, 2021, With Relevant Healthcare Common Procedure Coding Codes Used for Identification of Claims

eTable 2. *ICD-9* and *ICD-10* Code Prefixes Used to Group Claims by Clinical Category for Analysis Models

eTable 3. Models of Annual Out-of-Pocket Spending in the 4 Years Before and After Biosimilar Entry

eTable 4. Models of Out-of-Pocket Spending per Claim in the Period After Biosimilars Were Available

eTable 5. Sensitivity Analyses: Combined Effects Models Without Age and Sex

This supplemental material has been provided by the authors to give readers additional information about their work.

eTable 1. List of All Biologic Drugs and Biosimilar Formulations Available Prior to January 1, 2021, With Relevant Healthcare Common Procedure Coding Codes Used for Identification of Claims

Reference Biologic (generic name)	Biosimilar (generic pre-/suffix)	US Market Entry	HCPCS Codes
**Neupogen (filgrastim)**	--	Feb 1991	J1440 (closed 12/31/13)<br>J1441 (closed 12/31/13)<br>J1442
	Granix (tbo-)	Nov 2013	J1446 (closed 12/31/15)<br>J1447
	Zarxio (-sndz)	Sep 2015	Q5101
	Nivestym (-aafi)	Oct 2018	Q5110
**Remicade (infliximab)**	--	Aug 1998	J1745
	Inflectra (-dyyb)	Nov 2016	Q5102 (closed 3/31/18)<br>Q5103
	Renflexis (-abda)	Jul 2017	Q5102 (closed 3/31/18)<br>Q5104
	Avsola (-axxq)	Jul 2020	Q5121
**Neulasta (pegfilgrastim)**	--	Jan 2002	J2505 (closed 12/31/21)<br>J2506 (after 1/1/22)
	Fulphila (-jmdb)	Jul 2018	Q5108
	Udenyca (-cbqv)	Jan 2019	Q5111
	Ziextenzo (-bmez)	Nov 2019	Q5120
**Epogen (epoetin alfa)**	--	June 1989	J0886 (closed 12/31/15)<br>Q4081<br>J0885
	Retacrit (-epbx)	Nov 2018	Q5105<br>Q5106
**Avastin (bevacizumab)**	--	Feb 2004	J9035
	Mvasi (-awwb)	Jul 2019	Q5107
	Zirabev (-bvzr)	Jan 2020	Q5118
**Rituxan (rituximab)**	--	Nov 1997	J9310 (closed 12/31/18)<br>J9312
	Truxima (-abbs)	Nov 2019	Q5115
	Ruxience (-pvvr)	Jan 2020	Q5119
	Riabni (-arrx)	Jan 2021	Q5123

**Herceptin (trastuzumab)**	--	Sep 1998	J9355
	Kanjinti (-anns)	Jul 2019	Q5117
	Ogivri (-dkst)	Dec 2019	Q5114
	Trazimera (-qyyp)	Feb 2020	Q5116
	Herzuma (-pkrb)	Mar 2020	Q5113
	Ontruzant (-dttb)	Apr 2020	Q5112

eTable 2. *ICD-9* and *ICD-10* Code Prefixes Used to Group Claims by Clinical Category for Analysis Models

Clinical Category	ICD-9 prefix	ICD-10 prefix	HCPCS Codes
**Hematology**	279-289	D50-89	Diseases of the blood and immune system
**Oncology**	140-239	C<br>D00-D49	Neoplasms
		Z51	Encounters for chemotherapy
**Rheumatology**	680-709	L	Diseases of the skin and subcutaneous tissue
	710-739	M	Diseases of the musculoskeletal system and connective tissue
	446	I70-I79	Diseases of arteries
**Nephrology**	580-629	N	Diseases of the genitourinary system
**Gastroenterology**	520-579	K	Diseases of the digestive system
**Ophthalmology**	250	E	Diabetes mellitus (with ophthalmologic manifestations)
	360-379	H	Diseases of the eye
**Neurology**	320-359	G	Diseases of the nervous system

eTable 3. Models of Annual Out-of-Pocket Spending in the 4 Years Before and After Biosimilar Entry

A. **Combined effects model with all drugs included**

		All drugs					
		**Model of Nonzero OOP Costs** (N = 251,566)			**Model of Mean Nonzero OOP Costs** (N = 122,784)		
		Unadjusted % Nonzero OOP	Adjusted Odds Ratio (95% CI)	p-value	Unadjusted Mean Nonzero OOP ($)	Adjusted Mean Ratio (95% CI)	p-value
**Years Since Biosimilar Entry**	-4	43.3%	0.87 (0.84, 0.90)	<0.001	$1482	0.80 (0.79, 0.82)	<0.001
	-3	45.1%	0.91 (0.88, 0.94)	<0.001	$1587	0.88 (0.86, 0.90)	<0.001
	-2	45.6%	0.92 (0.89, 0.95)	<0.001	$1638	0.94 (0.92, 0.96)	<0.001
	-1	48.5%	Ref	Ref	$1704	Ref	Ref
	0	49.8%	1.02 (0.98, 1.05)	0.285	$1767	1.04 (1.02, 1.06)	<0.001
	1	50.5%	1.03 (1.00, 1.07)	0.074	$1890	1.08 (1.06, 1.10)	<0.001
	2	52.6%	1.08 (1.04, 1.12)	<0.001	$1953	1.12 (1.10, 1.14)	<0.001
	3	51.1%	1.15 (1.10, 1.20)	<0.001	$2712	1.15 (1.12, 1.18)	<0.001
	4	64.3%	1.22 (1.16, 1.29)	<0.001	$2984	1.18 (1.15, 1.21)	<0.001
**Age**	0-17	54.2%	Ref	Ref	$2270	Ref	Ref
	18-44	52.0%	1.20 (1.14, 1.26)	<0.001	$2101	1.06 (1.03, 1.08)	<0.001
	45-64	42.0%	1.17 (1.11, 1.23)	<0.001	$1495	1.08 (1.05, 1.10)	<0.001
**Sex**	Female	43.6%	Ref	Ref	$1732	Ref	Ref
	Male	47.6%	0.95 (0.94, 0.97)	<0.001	$1708	1.02 (1.01, 1.03)	<0.001
**US Census Region**	Northeast	39.1%	Ref	Ref	$1652	Ref	Ref
	Midwest	48.3%	1.45 (1.40, 1.50)	<0.001	$1809	1.11 (1.09, 1.12)	<0.001
	South	44.2%	1.29 (1.25, 1.33)	<0.001	$1700	1.19 (1.17, 1.21)	<0.001

	West	46.4%	1.33 (1.28, 1.38)	<0.001	$1667	1.11 (1.09, 1.13)	<0.001
**Site of Service**	Office	48.3%	Ref	Ref	$1608	Ref	Ref
	Outpatient Hospital	39.3%	0.83 (0.81, 0.84)	<0.001	$1811	0.97 (0.96, 0.98)	<0.001
	Other	46.6%	0.88 (0.84, 0.92)	<0.001	$2639	1.24 (1.22, 1.27)	<0.001
**High-Deductible Health Plan**	No	42.8%	Ref	Ref	$1590	Ref	Ref
	Yes	53.4%	1.48 (1.45, 1.51)	<0.001	$2072	1.19 (1.18, 1.20)	<0.001
**Primary Disease Category**	Gastro-intestinal	69.9%	Ref	Ref	$2633	Ref	Ref
	Hematologic	24.9%	0.44 (0.41, 0.48)	<0.001	$887	1.03 (0.99, 1.08)	0.161
	Oncologic	29.7%	0.37 (0.35, 0.39)	<0.001	$1549	0.93 (0.90, 0.96)	<0.001
	Ophthalmo-logic	60.8%	1.07 (1.00, 1.15)	0.049	$140	0.06 (0.06, 0.06)	<0.001
	Rheumato-logic	62.4%	0.90 (0.87, 0.93)	<0.001	$2584	1.07 (1.05, 1.08)	<0.001
	Renal	25.6%	0.51 (0.43, 0.60)	<0.001	$791	2.56 (2.36, 2.78)	<0.001
	Other	11.1%	0.56 (0.54, 0.60)	<0.001	$1819	0.87 (0.85, 0.89)	<0.001

B. Filgrastim

		Filgrastim					
		Model of Nonzero OOP Costs (N = 23,638)			Model of Mean Nonzero OOP Costs (N = 5,321)		
		Unadjusted % Nonzero OOP	Adjusted Odds Ratio (95% CI)	p-value	Unadjusted Mean Nonzero OOP ($)	Adjusted Mean Ratio (95% CI)	p-value
**Years Since Biosimilar Entry**	-4	22.2%	0.94 (0.83, 1.07)	0.344	$300	0.70 (0.60, 0.82)	<0.001
	-3	22.7%	0.97 (0.86, 1.11)	0.690	$359	0.84 (0.72, 0.97)	0.019
	-2	22.7%	0.96 (0.85, 1.09)	0.549	$390	0.91 (0.79, 1.05)	0.219
	-1	23.2%	Ref	Ref	$439	Ref	Ref
	0	23.5%	1.03 (0.91, 1.17)	0.633	$516	1.12 (0.96, 1.30)	0.147
	1	23.8%	1.06 (0.93, 1.21)	0.388	$543	1.23 (1.05, 1.46)	0.013
	2	23.1%	1.02 (0.90, 1.17)	0.729	$532	1.16 (0.99, 1.35)	0.061
	3	20.6%	0.91 (0.80, 1.04)	0.173	$621	1.43 (1.23, 1.66)	<0.001
	4	21.2%	0.95 (0.83, 1.09)	0.490	$656	1.44 (1.23, 1.68)	<0.001
**Age**	0-17	21.2%	Ref	Ref	$568	Ref	Ref
	18-44	22.5%	1.04 (0.87, 1.25)	0.633	$543	1.33 (0.99, 1.77)	0.055
	45-64	20.9%	0.96 (0.81, 1.14)	0.625	$556	1.44 (1.09, 1.91)	0.010
**Sex**	Female	22.8%	Ref	Ref	$566	Ref	Ref
	Male	19.0%	0.85 (0.81, 0.91)	<0.001	$531	1.00 (0.93, 1.08)	0.925
**US Census Region**	Northeast	18.1%	Ref	Ref	$471	Ref	Ref
	Midwest	20.6%	1.13 (0.99, 1.28)	0.070	$560	1.17 (1.00, 1.37)	0.055
	South	21.6%	1.16 (1.03, 1.31)	0.016	$569	1.20 (1.04, 1.40)	0.016
	West	22.7%	1.25 (1.10, 1.43)	0.001	$538	1.07 (0.90, 1.26)	0.447
**Site of Service**	Office	25.5%	Ref	Ref	$557	Ref	Ref
	Outpatient Hospital	17.2%	0.81 (0.76, 0.86)	<0.001	$509	0.92 (0.84, 0.99)	0.032

	Other	26.6%	1.07 (0.88, 1.31)	0.563	$1054	2.25 (1.69, 3.00)	<0.001
**High-Deductible Health Plan**	No	21.1%	Ref	Ref	$510	Ref	Ref
	Yes	21.6%	1.07 (1.00, 1.15)	0.037	$676	1.34 (1.22, 1.47)	<0.001
**Primary Disease Category**	Hematologic	26.3%	Ref	Ref	$611	Ref	Ref
	Oncologic	19.1%	0.74 (0.70, 0.79)	<0.001	$527	0.87 (0.82, 0.94)	<0.001
	Other	21.4%	1.00 (0.88, 1.14)	0.996	$454	0.71 (0.58, 0.86)	0.001

C. Infliximab

		Infliximab					
		Model of Nonzero OOP Costs (N = 79,038)			Model of Mean Nonzero OOP Costs (N = 56,077)		
		Unadjusted % Nonzero OOP	Adjusted Odds Ratio (95% CI)	p-value	Unadjusted Mean Nonzero OOP ($)	Adjusted Mean Ratio (95% CI)	p-value
**Years Since Biosimilar Entry**	-4	62.3%	0.73 (0.69, 0.78)	<0.001	$2150	0.83 (0.81, 0.86)	<0.001
	-3	66.0%	0.84 (0.79, 0.89)	<0.001	$2363	0.91 (0.89, 0.93)	<0.001
	-2	69.0%	0.96 (0.91, 1.01)	0.123	$2512	0.97 (0.94, 0.99)	0.007
	-1	70.1%	Ref	Ref	$2606	Ref	Ref
	0	70.6%	1.00 (0.95, 1.06)	0.956	$2619	1.00 (0.98, 1.02)	0.923
	1	71.4%	1.03 (0.97, 1.10)	0.288	$2855	1.09 (1.06, 1.11)	<0.001
	2	74.9%	1.21 (1.13, 1.29)	<0.001	$3087	1.16 (1.13, 1.19)	<0.001
	3	76.4%	1.30 (1.22, 1.39)	<0.001	$3140	1.18 (1.15, 1.21)	<0.001
	4	76.3%	1.26 (1.18, 1.35)	<0.001	$3151	1.17 (1.14, 1.21)	<0.001
**Age**	0-17	68.1%	Ref	Ref	$2661	Ref	Ref
	18-44	75.0%	1.30 (1.21, 1.40)	<0.001	$2931	1.04 (1.01, 1.07)	0.018
	45-64	73.7%	1.21 (1.12, 1.31)	<0.001	$2934	1.04 (1.01, 1.07)	0.018
**Sex**	Female	73.4%	Ref	Ref	$2828	Ref	Ref
	Male	74.5%	1.03 (0.99, 1.08)	0.162	$2999	1.07 (1.05, 1.09)	<0.001
**US Census Region**	Northeast	66.5%	Ref	Ref	$2505	Ref	Ref
	Midwest	79.1%	1.95 (1.81, 2.10)	<0.001	$2885	1.14 (1.10, 1.18)	<0.001
	South	71.6%	1.32 (1.23, 1.42)	<0.001	$3043	1.23 (1.19, 1.27)	<0.001
	West	74.5%	1.50 (1.39, 1.63)	<0.001	$2890	1.16 (1.12, 1.20)	<0.001
**Site of Service**	Office	75.4%	Ref	Ref	$2967	Ref	Ref
	Outpatient Hospital	66.8%	1.00 (0.95, 1.05)	0.995	$2515	0.93 (0.91, 0.95)	<0.001

	Other	80.0%	1.38 (1.27, 1.49)	<0.001	$3282	1.12 (1.09, 1.15)	<0.001
**High-Deductible Health Plan**	No	70.3%	Ref	Ref	$2713	Ref	Ref
	Yes	83.0%	2.40 (2.30, 2.52)	<0.001	$3337	1.27 (1.25, 1.30)	<0.001
**Primary Disease Category**	Gastro-intestinal	74.5%	Ref	Ref	$2908	Ref	Ref
	Rheumato-logic	74.3%	0.97 (0.92, 1.02)	0.291	$2954	1.05 (1.02, 1.07)	<0.001
	Other	62.8%	0.63 (0.58, 0.68)	<0.001	$2665	0.97 (0.94, 1.01)	0.177

D. Pegfilgrastim

		Pegfilgrastim					
		Model of Nonzero OOP Costs (N = 50,110)			Model of Mean Nonzero OOP Costs (N = 12,442)		
		Unadjusted % Nonzero OOP	Adjusted Odds Ratio (95% CI)	p-value	Unadjusted Mean Nonzero OOP ($)	Adjusted Mean Ratio (95% CI)	p-value
**Years Since Biosimilar Entry**	-4	27.3%	1.18 (1.09, 1.28)	<0.001	$1181	0.85 (0.79, 0.92)	<0.001
	-3	26.7%	1.15 (1.07, 1.25)	<0.001	$1375	0.98 (0.91, 1.06)	0.672
	-2	24.4%	1.02 (0.94, 1.10)	0.667	$1404	1.00 (0.93, 1.09)	0.905
	-1	24.0%	Ref	Ref	$1416	Ref	Ref
	0	23.6%	0.98 (0.90, 1.06)	0.596	$1631	1.16 (1.08, 1.26)	<0.001
	1	22.8%	0.94 (0.87, 1.03)	0.178	$1661	1.19 (1.10, 1.29)	<0.001
	2	23.7%	0.99 (0.91, 1.08)	0.849	$1733	1.24 (1.11, 1.37)	<0.001
	3	25.7%	1.11 (1.02, 1.21)	0.017	$1523	1.09 (1.01, 1.17)	0.024
**Age**	0-17	12.3%	Ref	Ref	$2377	Ref	Ref
	18-44	23.7%	1.92 (1.51, 2.43)	<0.001	$1569	0.73 (0.50, 1.06)	0.100
	45-64	24.1%	1.92 (1.51, 2.43)	<0.001	$1592	0.74 (0.51, 1.07)	0.112
**Sex**	Female	24.8%	Ref	Ref	$1578	Ref	Ref
	Male	21.9%	0.87 (0.83, 0.90)	<0.001	$1625	1.01 (0.97, 1.05)	0.622
**US Census Region**	Northeast	20.1%	Ref	Ref	$1404	Ref	Ref
	Midwest	22.5%	1.28 (1.19, 1.37)	<0.001	$1546	1.08 (1.00, 1.16)	0.041
	South	25.0%	1.33 (1.24, 1.43)	<0.001	$1682	1.19 (1.11, 1.28)	<0.001
	West	25.8%	1.49 (1.38, 1.60)	<0.001	$1521	1.04 (0.97, 1.12)	0.246
**Site of Service**	Office	28.0%	Ref	Ref	$1479	Ref	Ref
	Outpatient Hospital	20.8%	0.90 (0.87, 0.94)	<0.001	$1707	1.18 (1.13, 1.23)	<0.001

	Other	21.9%	1.00 (0.78, 1.27)	0.977	$1497	0.83 (0.65, 1.07)	0.161
**High-Deductible Health Plan**	No	24.2%	Ref	Ref	$1529	Ref	Ref
	Yes	23.4%	1.04 (1.00, 1.09)	0.032	$1741	1.17 (1.13, 1.22)	<0.001
**Primary Disease Category**	Hematologic	26.5%	Ref	Ref	$1615	Ref	Ref
	Oncologic	23.8%	1.01 (0.96, 1.06)	0.754	$1588	0.97 (0.92, 1.02)	0.199
	Other	17.7%	0.80 (0.69, 0.92)	0.002	$1615	0.91 (0.80, 1.03)	0.134

E. Epoetin alfa

		Epoetin alfa					
		Model of Nonzero OOP Costs (N = 8,912)			Model of Mean Nonzero OOP Costs (N = 2,446)		
		Unadjusted % Nonzero OOP	Adjusted Odds Ratio (95% CI)	p-value	Unadjusted Mean Nonzero OOP ($)	Adjusted Mean Ratio (95% CI)	p-value
**Years Since Biosimilar Entry**	-4	23.8%	0.90 (0.76, 1.06)	0.218	$528	0.67 (0.51, 0.88)	0.004
	-3	23.0%	0.79 (0.67, 0.94)	0.008	$605	0.82 (0.62, 1.08)	0.161
	-2	30.2%	0.82 (0.70, 0.98)	0.027	$505	0.72 (0.56, 0.92)	0.008
	-1	35.6%	Ref	Ref	$712	Ref	Ref
	0	33.0%	0.88 (0.73, 1.06)	0.184	$603	0.90 (0.70, 1.16)	0.406
	1	33.3%	0.88 (0.73, 1.06)	0.189	$617	0.89 (0.69, 1.16)	0.394
	2	27.5%	0.67 (0.55, 0.81)	<0.001	$498	0.78 (0.59, 1.03)	0.079
	3	31.4%	0.80 (0.66, 0.97)	0.021	$544	0.78 (0.60, 1.02)	0.066
**Age**	0-17	31.7%	Ref	Ref	$167	Ref	Ref
	18-44	32.8%	1.12 (0.84, 1.50)	0.453	$495	1.84 (1.29, 2.62)	0.001
	45-64	32.2%	1.06 (0.80, 1.41)	0.686	$649	1.99 (1.42, 2.80)	<0.001
**Sex**	Female	33.1%	Ref	Ref	$644	Ref	Ref
	Male	31.5%	0.95 (0.88, 1.03)	0.229	$561	0.86 (0.77, 0.95)	0.004
**US Census Region**	Northeast	27.2%	Ref	Ref	$508	Ref	Ref
	Midwest	30.2%	1.30 (1.09, 1.54)	0.003	$591	1.27 (1.03, 1.57)	0.024
	South	36.1%	1.44 (1.23, 1.69)	<0.001	$636	1.30 (1.09, 1.56)	0.004
	West	26.3%	1.51 (1.26, 1.80)	<0.001	$540	0.95 (0.76, 1.18)	0.646
**Site of Service**	Office	39.0%	Ref	Ref	$638	Ref	Ref
	Outpatient Hospital	34.2%	1.23 (1.11, 1.36)	<0.001	$548	0.85 (0.76, 0.94)	0.003
	Other	12.8%	0.25 (0.22, 0.29)	<0.001	$741	1.44 (1.20, 1.74)	<0.001

**High-Deductible Health Plan**	No	31.0%	Ref	Ref	$626	Ref	Ref
	Yes	36.4%	1.21 (1.11, 1.33)	<0.001	$545	1.04 (0.92, 1.17)	0.550
**Primary Disease Category**	Hematologic	29.9%	Ref	Ref	$678	Ref	Ref
	Renal	35.5%	1.45 (1.31, 1.61)	<0.001	$637	0.96 (0.86, 1.08)	0.521
	Other	25.2%	0.74 (0.64, 0.85)	<0.001	$200	0.43 (0.36, 0.52)	<0.001

F. Bevacizumab

		Bevacizumab					
		Model of Nonzero OOP Costs (N = 51,875)			Model of Mean Nonzero OOP Costs (N = 29,809)		
		Unadjusted % Nonzero OOP	Adjusted Odds Ratio (95% CI)	p-value	Unadjusted Mean Nonzero OOP ($)	Adjusted Mean Ratio (95% CI)	p-value
**Years Since Biosimilar Entry**	-4	51.3%	0.81 (0.75, 0.87)	<0.001	$559	0.77 (0.73, 0.81)	<0.001
	-3	53.6%	0.84 (0.79, 0.90)	<0.001	$514	0.82 (0.79, 0.86)	<0.001
	-2	53.7%	0.81 (0.76, 0.86)	<0.001	$480	0.92 (0.89, 0.96)	<0.001
	-1	59.3%	Ref	Ref	$471	Ref	Ref
	0	60.1%	1.06 (0.99, 1.12)	0.074	$488	1.07 (1.03, 1.11)	<0.001
	1	60.3%	1.07 (1.00, 1.14)	0.047	$469	1.07 (1.03, 1.11)	0.001
	2	62.0%	1.12 (1.05, 1.20)	<0.001	$422	1.08 (1.04, 1.13)	<0.001
**Age**	0-17	40.3%	Ref	Ref	$797	Ref	Ref
	18-44	60.7%	1.32 (1.03, 1.70)	0.030	$390	1.16 (0.98, 1.37)	0.080
	45-64	60.5%	1.26 (0.98, 1.61)	0.074	$476	1.28 (1.08, 1.51)	0.004
**Sex**	Female	59.3%	Ref	Ref	$563	Ref	Ref
	Male	61.4%	1.01 (0.98, 1.05)	0.465	$382	1.00 (0.97, 1.03)	0.859
**US Census Region**	Northeast	50.9%	Ref	Ref	$484	Ref	Ref
	Midwest	62.2%	1.59 (1.47, 1.71)	<0.001	$421	1.04 (0.98, 1.11)	0.149
	South	62.2%	1.45 (1.35, 1.56)	<0.001	$488	1.12 (1.06, 1.19)	<0.001
	West	57.5%	1.28 (1.18, 1.39)	<0.001	$453	1.09 (1.02, 1.16)	0.007
**Site of Service**	Office	65.7%	Ref	Ref	$321	Ref	Ref
	Outpatient Hospital	34.5%	0.81 (0.76, 0.86)	<0.001	$1867	1.05 (0.97, 1.15)	0.243
	Other	16.4%	0.22 (0.16, 0.29)	<0.001	$418	1.10 (0.89, 1.36)	0.361
	No	58.1%	Ref	Ref	$454	Ref	Ref

**High-Deductible Health Plan**	Yes	66.8%	1.82 (1.75, 1.89)	<0.001	$480	1.06 (1.03, 1.09)	<0.001
**Primary Disease Category**	Oncologic	37.4%	Ref	Ref	$2070	Ref	Ref
	Ophthalmo-logic	68.0%	2.61 (2.49, 2.74)	<0.001	$170	0.07 (0.07, 0.07)	<0.001
	Other	53.0%	1.67 (1.44, 1.94)	<0.001	$762	0.36 (0.30, 0.44)	<0.001

G. Rituximab

		Rituximab					
		Model of Nonzero OOP Costs (N = 24,556)			Model of Mean Nonzero OOP Costs (N = 11,496)		
		Unadjusted % Nonzero OOP	Adjusted Odds Ratio (95% CI)	p-value	Unadjusted Mean Nonzero OOP ($)	Adjusted Mean Ratio (95% CI)	p-value
**Years Since Biosimilar Entry**	-4	44.1%	0.93 (0.84, 1.02)	0.122	$1997	0.80 (0.76, 0.85)	<0.001
	-3	44.3%	0.92 (0.84, 1.01)	0.081	$2175	0.87 (0.83, 0.93)	<0.001
	-2	45.5%	0.93 (0.85, 1.02)	0.106	$2348	0.93 (0.88, 0.97)	0.002
	-1	47.5%	Ref	Ref	$2545	Ref	Ref
	0	48.9%	1.03 (0.95, 1.12)	0.465	$2600	1.01 (0.97, 1.06)	0.594
	1	48.6%	1.02 (0.93, 1.12)	0.617	$2621	1.02 (0.97, 1.07)	0.446
	2	48.5%	1.01 (0.92, 1.10)	0.900	$2374	0.92 (0.87, 0.98)	0.005
**Age**	0-17	32.6%	Ref	Ref	$2046	Ref	Ref
	18-44	49.5%	1.72 (1.45, 2.03)	<0.001	$2529	1.09 (0.98, 1.22)	0.121
	45-64	48.5%	1.84 (1.56, 2.17)	<0.001	$2552	1.07 (0.96, 1.20)	0.202
**Sex**	Female	53.0%	Ref	Ref	$2618	Ref	Ref
	Male	41.9%	0.91 (0.86, 0.96)	<0.001	$2393	0.99 (0.96, 1.02)	0.548
**US Census Region**	Northeast	43.0%	Ref	Ref	$2170	Ref	Ref
	Midwest	45.5%	1.14 (1.04, 1.26)	0.007	$2370	1.06 (0.98, 1.15)	0.133
	South	49.7%	1.26 (1.15, 1.38)	<0.001	$2722	1.16 (1.08, 1.26)	<0.001
	West	52.1%	1.35 (1.22, 1.50)	<0.001	$2540	1.10 (1.02, 1.19)	0.016
**Site of Service**	Office	55.2%	Ref	Ref	$2832	Ref	Ref
	Outpatient Hospital	42.8%	0.97 (0.92, 1.02)	0.192	$2223	0.86 (0.83, 0.89)	<0.001
	Other	43.0%	0.61 (0.47, 0.81)	<0.001	$2471	0.97 (0.81, 1.16)	0.741
	No	48.0%	Ref	Ref	$2501	Ref	Ref

**High-Deductible Health Plan**	Yes	49.1%	1.31<br>(1.24, 1.39)	<0.001	$2620	1.14<br>(1.10, 1.18)	<0.001
**Primary Disease Category**	Oncologic	35.6%	Ref	Ref	$2155	Ref	Ref
	Rheumato-logic	62.4%	2.22<br>(2.09, 2.36)	<0.001	$2910	1.26<br>(1.21, 1.30)	<0.001
	Other	48.3%	1.42<br>(1.32, 1.52)	<0.001	$2238	1.03<br>(0.98, 1.08)	0.219

H. Trastuzumab

		Trastuzumab					
		**Model of Nonzero OOP Costs** (N = 13,437)			**Model of Mean Nonzero OOP Costs** (N = 5,193)		
		Unadjusted % Nonzero OOP	Adjusted Odds Ratio (95% CI)	p-value	Unadjusted Mean Nonzero OOP ($)	Adjusted Mean Ratio (95% CI)	p-value
**Years Since Biosimilar Entry**	-4	41.7%	1.18 (1.05, 1.33)	0.005	$1980	0.92 (0.84, 1.01)	0.082
	-3	39.6%	1.07 (0.95, 1.20)	0.241	$1979	0.92 (0.81, 1.04)	0.193
	-2	38.3%	1.03 (0.92, 1.15)	0.639	$2089	0.96 (0.88, 1.05)	0.331
	-1	37.6%	Ref	Ref	$2182	Ref	Ref
	0	37.5%	1.01 (0.90, 1.13)	0.932	$2176	1.02 (0.93, 1.11)	0.729
	1	34.4%	0.88 (0.78, 0.99)	0.032	$1961	0.91 (0.83, 1.00)	0.042
	2	35.3%	0.91 (0.81, 1.03)	0.136	$1913	0.88 (0.81, 0.96)	0.006
**Age**	18-44	33.2%	Ref	Ref	$1967	Ref	Ref
	45-64	37.3%	1.18 (1.06, 1.32)	0.002	$2103	1.08 (0.99, 1.19)	0.084
**Sex**	Female	36.4%	Ref	Ref	$2056	Ref	Ref
	Male	33.2%	0.76 (0.64, 0.89)	0.001	$2479	0.99 (0.84, 1.17)	0.943
**US Census Region**	Northeast	30.1%	Ref	Ref	$1462	Ref	Ref
	Midwest	35.2%	1.43 (1.28, 1.60)	<0.001	$2078	1.10 (0.89, 1.36)	0.393
	South	38.4%	1.47 (1.32, 1.64)	<0.001	$2147	1.15 (0.93, 1.42)	0.203
	West	36.2%	1.38 (1.22, 1.56)	<0.001	$2145	1.14 (0.92, 1.42)	0.231
**Site of Service**	Office	43.4%	Ref	Ref	$2149	Ref	Ref
	Outpatient Hospital	31.2%	0.85 (0.80, 0.90)	<0.001	$1999	0.96 (0.91, 1.02)	0.238
	Other	20.8%	0.32 (0.18, 0.56)	<0.001	$1123	0.64 (0.37, 1.10)	0.106
**High-Deductible Health Plan**	No	35.7%	Ref	Ref	$2056	Ref	Ref
	Yes	37.6%	1.34 (1.26, 1.43)	<0.001	$2109	1.19 (1.12, 1.26)	<0.001

**Primary Disease Category**	Oncologic	36.4%	Ref	Ref	$2069	Ref	Ref
	Other	31.3%	0.85 (0.66, 1.10)	0.212	$2431	1.19 (1.12, 1.26)	<0.001

eTable 4. Models of Out-of-Pocket Spending per Claim in the Period After Biosimilars Were Available

A. Combined effects model with all drugs included

		All Drugs					
		**Model of Nonzero OOP Costs** (N = 586,493)			**Model of Mean Nonzero OOP Costs** (N = 149,701)		
		Unadjusted % Nonzero OOP	Adjusted Odds Ratio (95% CI)	p-value	Unadjusted Mean Nonzero OOP ($)	Adjusted Mean Ratio (95% CI)	p-value
**Drug Type**	Reference Biologic	28.5%	Ref	Ref	$911	Ref	Ref
	Biosimilar	16.9%	1.13 (1.11, 1.16)	<0.001	$707	0.92 (0.90, 0.93)	<0.001
**Age**	0-17	24.4%	Ref	Ref	$1120	Ref	Ref
	18-44	30.2%	1.25 (1.21, 1.29)	<0.001	$1039	1.15 (1.12, 1.18)	<0.001
	45-64	23.1%	1.13 (1.09, 1.17)	<0.001	$740	1.12 (1.09, 1.15)	<0.001
**Sex**	Female	23.5%	Ref	Ref	$868	Ref	Ref
	Male	28.3%	0.93 (0.91, 0.94)	<0.001	$887	1.07 (1.06, 1.08)	<0.001
**US Census Region**	Northeast	21.1%	Ref	Ref	$897	Ref	Ref
	Midwest	26.1%	1.23 (1.20, 1.26)	<0.001	$920	1.06 (1.04, 1.08)	<0.001
	South	25.6%	1.30 (1.27, 1.33)	<0.001	$877	1.18 (1.16, 1.20)	<0.001
	West	26.5%	1.39 (1.35, 1.43)	<0.001	$801	1.04 (1.02, 1.06)	<0.001
**Site of Service**	Office	31.8%	Ref	Ref	$750	Ref	Ref
	Outpatient Hospital	13.3%	0.52 (0.51, 0.53)	<0.001	$1212	1.35 (1.33, 1.37)	<0.001
	Other	29.8%	0.71 (0.69, 0.73)	<0.001	$1255	1.31 (1.29, 1.33)	<0.001
**High-Deductible Health Plan**	No	25.3%	Ref	Ref	$807	Ref	Ref
	Yes	26.0%	1.07 (1.05, 1.08)	<0.001	$1039	1.17 (1.16, 1.18)	<0.001
**Primary Disease Category**	Gastro-intestinal	36.1%	Ref	Ref	$1167	Ref	Ref
	Hematologic	15.2%	0.47	<0.001	$235	0.75	<0.001

			(0.44, 0.50)			(0.71, 0.79)	
	Oncologic	9.0%	0.33 (0.31, 0.35)	<0.001	$828	0.83 (0.79, 0.87)	<0.001
	Ophthalmo-logic	58.3%	5.22 (4.85, 5.61)	<0.001	$61	0.07 (0.06, 0.07)	<0.001
	Rheumato-logic	35.7%	0.90 (0.89, 0.92)	<0.001	$1209	1.05 (1.04, 1.07)	<0.001
	Renal	19.3%	0.70 (0.64, 0.78)	<0.001	$146	0.63 (0.58, 0.68)	<0.001
	Other	22.4%	0.66 (0.63, 0.69)	<0.001	$1067	0.92 (0.89, 0.94)	<0.001
**Month**	January	60.8%	14.71 (14.23, 15.21)	<0.001	$1520	2.00 (1.95, 2.05)	<0.001
	February	45.5%	6.97 (6.74, 7.21)	<0.001	$1205	1.56 (1.52, 1.60)	<0.001
	March	35.5%	4.01 (3.88, 4.14)	<0.001	$797	1.10 (1.08, 1.13)	<0.001
	April	29.5%	2.94 (2.84, 3.04)	<0.001	$716	1.02 (1.00, 1.05)	0.101
	May	25.0%	2.20 (2.13, 2.28)	<0.001	$664	0.95 (0.93, 0.98)	<0.001
	June	22.7%	1.87 (1.81, 1.94)	<0.001	$627	0.94 (0.92, 0.97)	<0.001
	July	20.3%	1.62 (1.57, 1.68)	<0.001	$623	0.99 (0.96, 1.02)	0.453
	August	18.3%	1.37 (1.32, 1.42)	<0.001	$578	0.93 (0.91, 0.96)	<0.001
	September	16.8%	1.22 (1.18, 1.26)	<0.001	$591	0.97 (0.94, 1.00)	0.031
	October	15.9%	1.13 (1.09, 1.17)	<0.001	$559	0.95 (0.92, 0.98)	<0.001
	November	14.5%	0.99 (0.96, 1.03)	0.589	$606	0.96 (0.93, 0.98)	0.001
	December	14.7%	Ref	Ref	$641	Ref	Ref
**Year**	2013	1.6%	Ref	Ref	$99	Ref	Ref
	2014	14.2%	0.64 (0.56, 0.74)	<0.001	$159	1.26 (1.11, 1.42)	<0.001
	2015	13.3%	0.60 (0.53, 0.69)	<0.001	$170	1.24 (1.10, 1.40)	0.001
	2016	13.2%	0.50 (0.44, 0.57)	<0.001	$515	1.43 (1.27, 1.61)	<0.001
	2017	27.8%	0.47 (0.41, 0.53)	<0.001	$1108	1.35 (1.20, 1.52)	<0.001
	2018	25.3%	0.50 (0.44, 0.57)	<0.001	$1183	1.40 (1.24, 1.57)	<0.001

	2019	23.9%	0.56 (0.49, 0.64)	<0.001	$947	1.32 (1.17, 1.49)	<0.001
	2020	26.6%	0.58 (0.51, 0.66)	<0.001	$849	1.22 (1.09, 1.38)	0.001
	2021	29.5%	0.66 (0.58, 0.75)	<0.001	$710	1.14 (1.01, 1.28)	0.032

B. Filgrastim

		Filgrastim					
		Model of Nonzero OOP Costs (N = 103,786)			Model of Mean Nonzero OOP Costs (N = 12,825)		
		Unadjusted % Nonzero OOP	Adjusted Odds Ratio (95% CI)	p-value	Unadjusted Mean Nonzero OOP ($)	Adjusted Mean Ratio (95% CI)	p-value
**Drug Type**	Reference Biologic	13.0%	Ref	Ref	$190	Ref	Ref
	Biosimilar	11.7%	1.01 (0.87, 1.17)	0.893	$150	0.79 (0.71, 0.88)	<0.001
**Age**	0-17	10.0%	Ref	Ref	$206	Ref	Ref
	18-44	12.6%	1.39 (0.95, 2.04)	0.087	$187	1.70 (1.17, 2.48)	0.005
	45-64	12.4%	1.30 (0.89, 1.91)	0.174	$166	1.57 (1.10, 2.24)	0.014
**Sex**	Female	13.5%	Ref	Ref	$165	Ref	Ref
	Male	10.4%	0.76 (0.67, 0.86)	<0.001	$184	1.08 (1.00, 1.17)	0.054
**US Census Region**	Northeast	11.3%	Ref	Ref	$169	Ref	Ref
	Midwest	12.0%	0.95 (0.65, 1.40)	0.802	$178	1.12 (0.92, 1.35)	0.260
	South	12.1%	0.94 (0.64, 1.37)	0.746	$178	1.20 (1.01, 1.42)	0.033
	West	13.7%	1.10 (0.74, 1.64)	0.624	$151	1.02 (0.86, 1.21)	0.839
**Site of Service**	Office	14.8%	Ref	Ref	$138	Ref	Ref
	Outpatient Hospital	8.8%	0.59 (0.51, 0.68)	<0.001	$235	1.67 (1.51, 1.84)	<0.001
	Other	13.0%	0.84 (0.56, 1.28)	0.424	$342	2.61 (1.76, 3.87)	<0.001
**High-Deductible Health Plan**	No	12.2%	Ref	Ref	$160	Ref	Ref
	Yes	12.7%	1.06 (0.91, 1.25)	0.435	$198	1.24 (1.12, 1.37)	<0.001
**Primary Disease Category**	Hematologic	16.1%	Ref	Ref	$152	Ref	Ref
	Oncologic	10.3%	0.66 (0.58, 0.75)	<0.001	$185	1.06 (0.98, 1.15)	0.146
	Other	13.0%	0.90 (0.68, 1.19)	0.469	$210	1.19 (0.97, 1.45)	0.096
**Month**	January	47.2%	15.89	<0.001	$248	1.42 (1.21, 1.67)	<0.001

			(13.13, 19.24)				
	February	27.6%	6.57 (5.41, 7.97)	<0.001	$137	0.80 (0.67, 0.94)	0.008
	March	15.7%	3.14 (2.57, 3.85)	<0.001	$122	0.73 (0.61, 0.87)	<0.001
	April	11.3%	2.13 (1.71, 2.65)	<0.001	$131	0.78 (0.64, 0.95)	0.012
	May	8.2%	1.49 (1.19, 1.85)	<0.001	$146	0.80 (0.65, 0.98)	0.033
	June	7.8%	1.40 (1.13, 1.74)	0.002	$155	0.93 (0.75, 1.16)	0.542
	July	7.5%	1.35 (1.07, 1.70)	0.012	$177	1.07 (0.86, 1.32)	0.535
	August	6.2%	1.07 (0.85, 1.34)	0.577	$129	0.76 (0.62, 0.94)	0.011
	September	5.3%	0.92 (0.73, 1.17)	0.491	$153	0.90 (0.70, 1.15)	0.394
	October	5.7%	1.00 (0.79, 1.26)	0.991	$146	0.85 (0.70, 1.03)	0.106
	November	5.7%	0.94 (0.77, 1.14)	0.507	$144	0.99 (0.80, 1.22)	0.928
	December	6.1%	Ref	Ref	$151	Ref	Ref
**Year**	2013	8.4%	Ref	Ref	$99	Ref	Ref
	2014	14.2%	0.73 (0.49, 1.09)	0.121	$159	1.65 (1.21, 2.26)	0.002
	2015	13.3%	0.68 (0.46, 1.01)	0.057	$170	1.72 (1.25, 2.36)	0.001
	2016	11.7%	0.64 (0.43, 0.96)	0.032	$191	2.04 (1.50, 2.79)	<0.001
	2017	11.9%	0.61 (0.40, 0.92)	0.017	$213	2.30 (1.66, 3.19)	<0.001
	2018	12.3%	0.62 (0.40, 0.95)	0.030	$169	1.79 (1.28, 2.51)	0.001
	2019	11.4%	0.61 (0.40, 0.93)	0.022	$170	1.90 (1.37, 2.64)	<0.001
	2020	11.8%	0.62 (0.40, 0.96)	0.032	$167	1.88 (1.32, 2.69)	0.001
	2021	13.2%	0.75 (0.48, 1.15)	0.189	$131	1.52 (1.09, 2.12)	0.014

C. Infliximab

		Infliximab					
		Model of Nonzero OOP Costs (N = 253,669)			Model of Mean Nonzero OOP Costs (N = 90,432)		
		Unadjusted % Nonzero OOP	Adjusted Odds Ratio (95% CI)	p-value	Unadjusted Mean Nonzero OOP ($)	Adjusted Mean Ratio (95% CI)	p-value
**Drug Type**	Reference Biologic	35.3%	Ref	Ref	$1213	Ref	Ref
	Biosimilar	37.9%	1.22 (1.15, 1.31)	<0.001	$813	1.00 (0.96, 1.03)	0.805
**Age**	0-17	27.2%	Ref	Ref	$1164	Ref	Ref
	18-44	37.5%	1.18 (1.09, 1.28)	<0.001	$1165	1.15 (1.10, 1.21)	<0.001
	45-64	35.3%	1.01 (0.93, 1.10)	0.829	$1151	1.13 (1.07, 1.19)	<0.001
**Sex**	Female	35.9%	Ref	Ref	$1108	Ref	Ref
	Male	35.4%	0.97 (0.93, 1.02)	0.194	$1214	1.09 (1.06, 1.12)	<0.001
**US Census Region**	Northeast	28.3%	Ref	Ref	$1106	Ref	Ref
	Midwest	35.8%	1.34 (1.23, 1.46)	<0.001	$1181	1.06 (1.00, 1.11)	0.036
	South	36.4%	1.30 (1.20, 1.41)	<0.001	$1190	1.18 (1.13, 1.25)	<0.001
	West	38.4%	1.55 (1.42, 1.70)	<0.001	$1080	1.03 (0.98, 1.08)	0.270
**Site of Service**	Office	39.7%	Ref	Ref	$1069	Ref	Ref
	Outpatient Hospital	24.5%	0.45 (0.43, 0.47)	<0.001	$1438	1.33 (1.28, 1.37)	<0.001
	Other	35.1%	0.77 (0.73, 0.82)	<0.001	$1300	1.29 (1.24, 1.34)	<0.001
**High-Deductible Health Plan**	No	35.5%	Ref	Ref	$1055	Ref	Ref
	Yes	35.9%	1.01 (0.97, 1.06)	0.567	$1398	1.22 (1.18, 1.25)	<0.001
**Primary Disease Category**	Gastro-intestinal	36.1%	Ref	Ref	$1167	Ref	Ref
	Rheum-atologic	35.9%	0.92 (0.87, 0.98)	0.008	$1135	1.04 (1.01, 1.07)	0.015
	Other	27.5%	0.73 (0.66, 0.80)	<0.001	$1186	0.99 (0.93, 1.05)	0.693
**Month**	January	72.6%	13.14	<0.001	$2045	2.13	<0.001

			(12.42, 13.92)			(2.04, 2.23)	
	February	64.3%	8.82 (8.36, 9.30)	<0.001	$1647	1.73 (1.65, 1.81)	<0.001
	March	52.6%	5.24 (4.99, 5.51)	<0.001	$989	1.06 (1.02, 1.11)	0.009
	April	45.0%	3.78 (3.60, 3.97)	<0.001	$886	0.96 (0.92, 1.01)	0.083
	May	37.0%	2.70 (2.57, 2.83)	<0.001	$801	0.87 (0.83, 0.91)	<0.001
	June	32.6%	2.18 (2.08, 2.28)	<0.001	$769	0.84 (0.80, 0.88)	<0.001
	July	29.0%	1.81 (1.73, 1.90)	<0.001	$834	0.90 (0.86, 0.94)	<0.001
	August	25.4%	1.51 (1.45, 1.58)	<0.001	$825	0.89 (0.84, 0.94)	<0.001
	September	22.6%	1.28 (1.23, 1.34)	<0.001	$821	0.90 (0.85, 0.95)	<0.001
	October	20.5%	1.13 (1.09, 1.18)	<0.001	$824	0.89 (0.85, 0.94)	<0.001
	November	18.6%	1.03 (0.99, 1.08)	0.131	$854	0.91 (0.87, 0.96)	<0.001
	December	18.3%	Ref	Ref	$933	Ref	Ref
**Year**	2016	16.1%	Ref	Ref	$956	Ref	Ref
	2017	32.2%	0.96 (0.89, 1.03)	0.206	$1199	0.95 (0.89, 1.02)	0.190
	2018	33.3%	1.01 (0.94, 1.09)	0.768	$1292	1.01 (0.94, 1.09)	0.705
	2019	35.1%	1.07 (0.99, 1.15)	0.086	$1260	0.98 (0.91, 1.05)	0.580
	2020	38.6%	1.26 (1.16, 1.36)	<0.001	$1139	0.88 (0.82, 0.95)	0.001
	2021	42.5%	1.35 (1.24, 1.48)	<0.001	$964	0.78 (0.72, 0.84)	<0.001

D. Pegfilgrastim

		Pegfilgrastim					
		Model of Nonzero OOP Costs (N = 72,818)			Model of Mean Nonzero OOP Costs (N = 6,379)		
		Unadjusted % Nonzero OOP	Adjusted Odds Ratio (95% CI)	p-value	Unadjusted Mean Nonzero OOP ($)	Adjusted Mean Ratio (95% CI)	p-value
**Drug Type**	Reference Biologic	8.9%	Ref	Ref	$1178	Ref	Ref
	Biosimilar	7.4%	0.97 (0.84, 1.12)	0.679	$1044	0.99 (0.89, 1.10)	0.846
**Age**	0-17	4.0%	Ref	Ref	$1447	Ref	Ref
	18-44	8.8%	1.89 (1.19, 3.00)	0.007	$1130	0.77 (0.51, 1.18)	0.233
	45-64	8.8%	1.82 (1.15, 2.88)	0.010	$1179	0.80 (0.52, 1.21)	0.288
**Sex**	Female	9.3%	Ref	Ref	$1148	Ref	Ref
	Male	7.5%	0.79 (0.73, 0.86)	<0.001	$1224	1.06 (0.98, 1.15)	0.149
**US Census Region**	Northeast	6.5%	Ref	Ref	$1046	Ref	Ref
	Midwest	7.9%	1.06 (0.91, 1.24)	0.424	$1181	1.10 (0.94, 1.29)	0.220
	South	9.5%	1.31 (1.13, 1.52)	<0.001	$1216	1.23 (1.09, 1.40)	0.001
	West	9.6%	1.29 (1.10, 1.51)	0.001	$1093	1.12 (0.99, 1.27)	0.072
**Site of Service**	Office	10.9%	Ref	Ref	$1021	Ref	Ref
	Outpatient Hospital	7.0%	0.56 (0.52, 0.60)	<0.001	$1361	1.29 (1.19, 1.40)	<0.001
	Other	5.8%	0.49 (0.27, 0.90)	0.021	$1115	1.02 (0.69, 1.52)	0.907
**High-Deductible Health Plan**	No	9.0%	Ref	Ref	$1120	Ref	Ref
	Yes	8.2%	0.89 (0.82, 0.97)	0.005	$1288	1.11 (1.01, 1.23)	0.035
**Primary Disease Category**	Hematologic	10.0%	Ref	Ref	$1067	Ref	Ref
	Oncologic	8.7%	0.93 (0.81, 1.07)	0.292	$1179	1.03 (0.93, 1.14)	0.585
	Other	6.2%	0.64 (0.46, 0.87)	0.004	$1134	1.01 (0.79, 1.29)	0.928
**Month**	January	46.1%	19.03	<0.001	$1567	1.60 (1.38, 1.85)	<0.001

			(16.61, 21.82)				
	February	17.4%	4.46 (3.85, 5.17)	<0.001	$873	0.93 (0.80, 1.08)	0.327
	March	8.0%	1.83 (1.55, 2.15)	<0.001	$791	0.83 (0.70, 0.99)	0.034
	April	6.1%	1.36 (1.14, 1.62)	0.001	$842	0.88 (0.74, 1.06)	0.173
	May	5.0%	1.10 (0.91, 1.31)	0.328	$780	0.84 (0.70, 1.01)	0.061
	June	4.5%	0.99 (0.82, 1.19)	0.895	$853	0.90 (0.75, 1.08)	0.253
	July	5.1%	1.10 (0.93, 1.30)	0.260	$1082	1.11 (0.89, 1.38)	0.339
	August	4.6%	0.98 (0.83, 1.17)	0.861	$972	1.00 (0.79, 1.27)	0.984
	September	4.5%	0.97 (0.81, 1.15)	0.697	$1196	1.21 (0.95, 1.55)	0.120
	October	4.9%	1.07 (0.91, 1.27)	0.423	$1065	1.08 (0.87, 1.34)	0.491
	November	3.8%	0.82 (0.69, 0.97)	0.020	$897	0.95 (0.80, 1.12)	0.531
	December	4.6%	Ref	Ref	$969	Ref	Ref
**Year**	2018	4.6%	Ref	Ref	$1047	Ref	Ref
	2019	9.1%	0.94 (0.81, 1.08)	0.349	$1208	0.98 (0.82, 1.18)	0.859
	2020	9.2%	0.95 (0.82, 1.10)	0.495	$1266	1.03 (0.83, 1.27)	0.797
	2021	10.1%	1.16 (1.00, 1.34)	0.046	$1066	0.88 (0.74, 1.05)	0.168

E. Epoetin alfa

		Epoetin alfa					
		Model of Nonzero OOP Costs (N = 16,188)			Model of Mean Nonzero OOP Costs (N = 2,895)		
		Unadjusted % Nonzero OOP	Adjusted Odds Ratio (95% CI)	p-value	Unadjusted Mean Nonzero OOP ($)	Adjusted Mean Ratio (95% CI)	p-value
**Drug Type**	Reference Biologic	15.5%	Ref	Ref	$171	Ref	Ref
	Biosimilar	21.2%	1.33 (0.97, 1.83)	0.075	$164	0.87 (0.67, 1.14)	0.323
**Age**	0-17	8.8%	Ref	Ref	$103	Ref	Ref
	18-44	18.9%	3.48 (1.60, 7.56)	0.002	$145	2.37 (1.26, 4.46)	0.007
	45-64	18.0%	3.24 (1.56, 6.72)	0.002	$174	2.64 (1.43, 4.89)	0.002
**Sex**	Female	20.4%	Ref	Ref	$168	Ref	Ref
	Male	15.8%	0.88 (0.67, 1.16)	0.370	$167	1.03 (0.87, 1.23)	0.706
**US Census Region**	Northeast	20.0%	Ref	Ref	$153	Ref	Ref
	Midwest	13.1%	1.39 (0.86, 2.25)	0.178	$167	1.17 (0.81, 1.69)	0.407
	South	21.4%	1.53 (1.00, 2.34)	0.049	$173	1.37 (0.98, 1.92)	0.068
	West	15.3%	1.22 (0.70, 2.13)	0.486	$154	1.24 (0.82, 1.89)	0.312
**Site of Service**	Office	25.3%	Ref	Ref	$156	Ref	Ref
	Outpatient Hospital	21.8%	0.87 (0.65, 1.17)	0.348	$199	1.29 (1.06, 1.56)	0.010
	Other	3.8%	0.08 (0.04, 0.17)	<0.001	$78	0.46 (0.27, 0.78)	0.004
**High-Deductible Health Plan**	No	16.9%	Ref	Ref	$166	Ref	Ref
	Yes	21.4%	0.94 (0.72, 1.23)	0.669	$173	1.02 (0.85, 1.21)	0.867
**Primary Disease Category**	Oncologic	13.1%	Ref	Ref	$234	Ref	Ref
	Renal	19.3%	3.60 (2.57, 5.04)	<0.001	$146	0.71 (0.56, 0.89)	0.004
	Other	18.0%	1.66 (1.20, 2.30)	0.002	$202	0.88 (0.68, 1.13)	0.312
**Month**	January	42.7%	9.40 (6.85, 12.90)	<0.001	$262	1.38 (0.94, 2.03)	0.102

	February	31.6%	5.22 (3.84, 7.10)	<0.001	$182	0.95 (0.63, 1.43)	0.811
	March	25.9%	3.79 (2.80, 5.13)	<0.001	$169	0.89 (0.60, 1.33)	0.571
	April	22.1%	2.84 v(2.10, 3.83)	<0.001	$141	0.78 (0.52, 1.17)	0.225
	May	19.1%	2.34 (1.71, 3.19)	<0.001	$134	0.71 (0.47, 1.08)	0.107
	June	15.8%	1.92 (1.42, 2.59)	<0.001	$146	0.79 (0.51, 1.23)	0.296
	July	13.8%	1.62 (1.21, 2.17)	0.001	$130	0.74 (0.49, 1.11)	0.141
	August	13.2%	1.51 (1.13, 2.01)	0.005	$134	0.81 (0.51, 1.30)	0.383
	September	13.0%	1.49 (1.13, 1.96)	0.004	$145	0.78 (0.51, 1.19)	0.249
	October	11.9%	1.29 (0.98, 1.69)	0.068	$144	0.79 (0.51, 1.23)	0.296
	November	8.8%	0.84 (0.65, 1.09)	0.189	$102	0.56 (0.38, 0.83)	0.004
	December	10.6%	Ref	Ref	$159	Ref	Ref
**Year**	2018	11.7%	Ref	Ref	$124	Ref	Ref
	2019	23.1%	0.92 (0.55, 1.53)	0.739	$178	1.00 (0.59, 1.68)	0.985
	2020	13.3%	0.44 (0.24, 0.83)	0.011	$175	1.04 (0.57, 1.88)	0.899
	2021	18.9%	0.58 (0.30, 1.12)	0.105	$156	0.99 (0.51, 1.92)	0.969

F. Bevacizumab

		Bevacizumab					
		Model of Nonzero OOP Costs (N = 77,308)			Model of Mean Nonzero OOP Costs (N = 29,608)		
		Unadjusted % Nonzero OOP	Adjusted Odds Ratio (95% CI)	p-value	Unadjusted Mean Nonzero OOP ($)	Adjusted Mean Ratio (95% CI)	p-value
**Drug Type**	Reference Biologic	48%	Ref	Ref	$104	Ref	Ref
	Biosimilar	8.7%	0.77 (0.68, 0.87)	<0.001	$1097	1.21 (1.00, 1.46)	0.048
**Age**	0-17	14.6%	Ref	Ref	$368	Ref	Ref
	18-44	39.2%	1.15 (0.76, 1.74)	0.517	$144	0.99 (0.72, 1.38)	0.969
	45-64	38.3%	1.15 (0.76, 1.73)	0.505	$162	0.96 (0.70, 1.33)	0.822
**Sex**	Female	35.4%	Ref	Ref	$197	Ref	Ref
	Male	41%	0.99 (0.91, 1.07)	0.775	$129	1.00 (0.97, 1.04)	0.938
**US Census Region**	Northeast	29.4%	Ref	Ref	$166	Ref	Ref
	Midwest	37.7%	1.27 (1.08, 1.50)	0.004	$135	0.95 (0.87, 1.04)	0.262
	South	40.1%	1.55 (1.32, 1.82)	<0.001	$181	1.05 (0.97, 1.15)	0.234
	West	38.8%	1.21 (1.01, 1.44)	0.037	$144	0.95 (0.87, 1.04)	0.309
**Site of Service**	Office	48.3%	Ref	Ref	$109	Ref	Ref
	Outpatient Hospital	7.4%	0.46 (0.40, 0.52)	<0.001	$1199	1.43 (1.25, 1.64)	<0.001
	Other	7.5%	0.06 (0.03, 0.12)	<0.001	$83	1.29 (1.01, 1.66)	0.040
**High-Deductible Health Plan**	No	37.2%	Ref	Ref	$158	Ref	Ref
	Yes	41.1%	1.44 (1.32, 1.56)	<0.001	$163	0.99 (0.95, 1.03)	0.554
**Primary Disease Category**	Oncologic	8.7%	Ref	Ref	$1146	Ref	Ref
	Ophthalmo-logic	58.3%	10.77 (9.52, 12.19)	<0.001	$61	0.09 (0.07, 0.10)	<0.001
	Other	24.3%	3.16 (2.44, 4.08)	<0.001	$315	0.31 (0.23, 0.43)	<0.001
**Month**	January	65.2%	8.71 (7.67, 9.88)	<0.001	$537	1.82 (1.73, 1.91)	<0.001

	February	52.5%	3.66 (3.31, 4.05)	<0.001	$208	1.56 (1.48, 1.64)	<0.001
	March	45.9%	2.56 (2.33, 2.81)	<0.001	$150	1.41 (1.34, 1.48)	<0.001
	April	42.1%	2.08 (1.91, 2.28)	<0.001	$120	1.30 (1.24, 1.37)	<0.001
	May	40.0%	1.81 (1.66, 1.98)	<0.001	$111	1.20 (1.15, 1.27)	<0.001
	June	38.2%	1.67 (1.54, 1.81)	<0.001	$98	1.17 (1.12, 1.23)	<0.001
	July	35.6%	1.44 (1.34, 1.55)	<0.001	$105	1.11 (1.06, 1.16)	<0.001
	August	34.2%	1.28 (1.19, 1.38)	<0.001	$77	1.04 (1.00, 1.09)	0.056
	September	33.5%	1.24 (1.16, 1.33)	<0.001	$112	1.07 (1.01, 1.13)	0.020
	October	32.9%	1.17 (1.09, 1.25)	<0.001	$89	1.04 (1.00, 1.09)	0.056
	November	30.0%	0.98 (0.92, 1.05)	0.513	$92	1.05 (1.00, 1.10)	0.044
	December	30.3%	Ref	Ref	$89	Ref	Ref
**Year**	2019	32.8%	Ref	Ref	$111	Ref	Ref
	2020	38.3%	0.97 (0.90, 1.04)	0.365	$183	0.97 (0.93, 1.01)	0.172
	2021	41.0%	1.08 (0.99, 1.18)	0.071	$158	0.94 (0.89, 0.98)	0.008

G. Rituximab

		Rituximab					
		Model of Nonzero OOP Costs (N = 22,229)			Model of Mean Nonzero OOP Costs (N = 4,997)		
		Unadjusted % Nonzero OOP	Adjusted Odds Ratio (95% CI)	p-value	Unadjusted Mean Nonzero OOP ($)	Adjusted Mean Ratio (95% CI)	p-value
**Drug Type**	Reference Biologic	23.9%	Ref	Ref	$1863	Ref	Ref
	Biosimilar	20.5%	1.12 (0.99, 1.26)	0.078	$1480	0.90 (0.82, 0.99)	0.022
**Age**	0-17	16.8%	Ref	Ref	$1827	Ref	Ref
	18-44	24.5%	1.63 (1.17, 2.27)	0.004	$1787	1.01 (0.83, 1.24)	0.889
	45-64	21.9%	1.58 (1.13, 2.19)	0.007	$1686	0.94 (0.77, 1.15)	0.578
**Sex**	Female	25.8%	Ref	Ref	$1770	Ref	Ref
	Male	18.2%	0.90 (0.81, 1.00)	0.053	$1616	1.01 (0.94, 1.08)	0.834
**US Census Region**	Northeast	19.3%	Ref	Ref	$1569	Ref	Ref
	Midwest	20.6%	0.96 (0.81, 1.15)	0.656	$1625	1.02 (0.90, 1.17)	0.739
	South	23.5%	1.12 (0.95, 1.33)	0.183	$1798	1.18 (1.04, 1.33)	0.009
	West	24.9%	1.20 (1.00, 1.45)	0.050	$1728	1.09 (0.96, 1.25)	0.180
**Site of Service**	Office	26.6%	Ref	Ref	$1723	Ref	Ref
	Outpatient Hospital	18.4%	0.73 (0.66, 0.81)	<0.001	$1708	1.06 (1.00, 1.14)	0.066
	Other	26.8%	0.66 (0.40, 1.07)	0.093	$1594	0.89 (0.70, 1.13)	0.325
**High-Deductible Health Plan**	No	23.0%	Ref	Ref	$1658	Ref	Ref
	Yes	21.3%	0.92 (0.83, 1.02)	0.113	$1862	1.10 (1.03, 1.18)	0.007
**Primary Disease Category**	Oncologic	13.5%	Ref	Ref	$1447	Ref	Ref
	Rheumato-logic	34.1%	3.56 (3.16, 4.00)	<0.001	$1895	1.39 (1.28, 1.50)	<0.001
	Other	25.2%	2.53 (2.21, 2.90)	<0.001	$1649	1.18 (1.07, 1.30)	0.001
**Month**	January	53.5%	11.11 (9.10, 13.56)	<0.001	$2073	1.55 (1.33, 1.80)	<0.001

	February	40.9%	5.98 (4.91, 7.28)	<0.001	$1882	1.35 (1.16, 1.58)	<0.001
	March	31.2%	3.74 (3.09, 4.54)	<0.001	$1838	1.30 (1.11, 1.52)	0.001
	April	25.7%	2.86 (2.34, 3.50)	<0.001	$1698	1.21 (1.03, 1.43)	0.023
	May	25.0%	2.56 (2.10, 3.14)	<0.001	$1803	1.30 (1.11, 1.53)	0.001
	June	24.2%	2.40 (1.99, 2.89)	<0.001	$1639	1.17 (1.00, 1.36)	0.050
	July	18.9%	1.82 (1.49, 2.23)	<0.001	$1580	1.13 (0.96, 1.33)	0.135
	August	15.8%	1.41 (1.15, 1.74)	0.001	$1391	1.01 (0.84, 1.21)	0.913
	September	14.1%	1.23 (0.99, 1.53)	0.056	$1526	1.06 (0.89, 1.25)	0.537
	October	13.0%	1.13 (0.92, 1.40)	0.246	$1378	1.00 (0.83, 1.20)	0.982
	November	12.3%	1.06 (0.88, 1.26)	0.554	$1373	0.99 (0.81, 1.20)	0.885
	December	11.7%	Ref	Ref	$1383	Ref	Ref
**Year**	2019	11.3%	Ref	Ref	$1480	Ref	Ref
	2020	23.1%	1.03 (0.84, 1.27)	0.746	$1859	1.00 (0.82, 1.23)	0.972
	2021	24.1%	1.07 (0.85, 1.35)	0.568	$1601	0.93 (0.76, 1.15)	0.529

H. Trastuzumab

		Trastuzumab					
		Model of Nonzero OOP Costs (N = 40,495)			Model of Mean Nonzero OOP Costs (N = 2,565)		
		Unadjusted % Nonzero OOP	Adjusted Odds Ratio (95% CI)	p-value	Unadjusted Mean Nonzero OOP ($)	Adjusted Mean Ratio (95% CI)	p-value
**Drug Type**	Reference Biologic	6.0%	Ref	Ref	$1309	Ref	Ref
	Biosimilar	6.6%	1.06 (0.90, 1.24)	0.477	$1062	0.92 (0.82, 1.02)	0.122
**Age**	18-44	5.2%	Ref	Ref	$1184	Ref	Ref
	45-64	6.7%	1.31 (1.12, 1.54)	0.001	$1152	1.00 (0.90, 1.12)	0.937
**Sex**	Female	6.3%	Ref	Ref	$1149	Ref	Ref
	Male	7.9%	1.35 (0.95, 1.94)	0.096	$1322	1.20 (0.87, 1.65)	0.262
**US Census Region**	Northeast	5.0%	Ref	Ref	$886	Ref	Ref
	Midwest	5.5%	0.93 (0.71, 1.21)	0.590	$1262	1.41 (1.18, 1.69)	<0.001
	South	6.9%	1.20 (0.92, 1.56)	0.172	$1155	1.44 (1.20, 1.73)	<0.001
	West	7.0%	1.18 (0.88, 1.58)	0.282	$1141	1.43 (1.17, 1.74)	<0.001
**Site of Service**	Office	8.0%	Ref	Ref	$1046	Ref	Ref
	Outpatient Hospital	5.0%	0.53 (0.46, 0.61)	<0.001	$1300	1.22 (1.09, 1.36)	<0.001
	Other	2.3%	0.21 (0.04, 1.16)	0.074	$796	0.70 (0.46, 1.07)	0.096
**High-Deductible Health Plan**	No	6.5%	Ref	Ref	$1129	Ref	Ref
	Yes	5.9%	0.89 (0.77, 1.03)	0.110	$1227	1.06 (0.96, 1.17)	0.261
**Primary Disease Category**	Oncologic	6.3%	Ref	Ref	$1154	Ref	Ref
	Other	6.9%	1.08 (0.75, 1.56)	0.682	$1419	1.14 (0.91, 1.43)	0.240
**Month**	January	41.9%	26.20 (20.14, 34.09)	<0.001	$1488	1.68 (1.37, 2.05)	<0.001
	February	17.2%	7.25 (5.52, 9.53)	<0.001	$821	0.94 (0.76, 1.16)	0.579
	March	7.2%	2.58	<0.001	$664	0.77	0.027

			(1.91, 3.50)			(0.61, 0.97)	
	April	4.1%	1.41<br>(1.02, 1.95)	0.036	$838	0.99<br>(0.72, 1.36)	0.950
	May	3.1%	1.06<br>(0.74, 1.52)	0.759	$898	1.04<br>(0.76, 1.44)	0.795
	June	2.3%	0.78<br>(0.54, 1.14)	0.201	$993	1.18<br>(0.84, 1.67)	0.344
	July	3.1%	1.11<br>(0.82, 1.49)	0.503	$1082	1.17<br>(0.88, 1.55)	0.274
	August	2.1%	0.73<br>(0.53, 1.01)	0.058	$803	0.85<br>(0.64, 1.13)	0.256
	September	2.3%	0.82<br>(0.60, 1.12)	0.202	$1532	1.53<br>(0.88, 2.68)	0.133
	October	2.4%	0.84<br>(0.62, 1.13)	0.253	$1002	1.14<br>(0.88, 1.47)	0.333
	November	2.2%	0.76<br>(0.57, 1.00)	0.052	$1082	1.26<br>(0.97, 1.63)	0.090
	December	2.9%	Ref	Ref	$900	Ref	Ref
**Year**	2019	2.4%	Ref	Ref	$1254	Ref	Ref
	2020	7.3%	0.97<br>(0.75, 1.26)	0.846	$1184	0.87<br>(0.65, 1.18)	0.371
	2021	7.7%	1.18<br>(0.89, 1.57)	0.248	$1115	0.87<br>(0.64, 1.18)	0.375

eTable 5. Sensitivity Analyses: Combined Effects Models Without Age and Sex

A. Annual OOP spending

		All drugs			
		**Model of Nonzero OOP Costs** (N =251,566)		**Model of Mean Nonzero OOP Costs** (N = 122,784)	
		Adjusted Odds Ratio (95% CI)	p-value	Adjusted Mean Ratio (95% CI)	p-value
**Years Since Biosimilar Entry**	-4	0.87 (0.85, 0.90)	<0.001	0.80 (0.79, 0.82)	<0.001
	-3	0.91 (0.88, 0.94)	<0.001	0.88 (0.86, 0.90)	<0.001
	-2	0.92 (0.89, 0.95)	<0.001	0.94 (0.92, 0.96)	<0.001
	-1	Ref	Ref	Ref	Ref
	0	1.02 (0.99, 1.05)	0.216	1.04 (1.02, 1.06)	<0.001
	1	1.04 (1.01, 1.08)	0.014	1.08 (1.06, 1.10)	<0.001
	2	1.10 (1.06, 1.14)	<0.001	1.12 (1.09, 1.14)	<0.001
	3	1.18 (1.14, 1.23)	<0.001	1.15 (1.12, 1.18)	<0.001
	4	1.34 (1.27, 1.40)	<0.001	1.18 (1.15, 1.21)	<0.001
**US Census Region**	Northeast	Ref	Ref	Ref	Ref
	Midwest	1.51 (1.47, 1.55)	<0.001	1.11 (1.09, 1.13)	<0.001
	South	1.39 (1.36, 1.43)	<0.001	1.18 (1.16, 1.20)	<0.001
	West	1.46 (1.42, 1.50)	<0.001	1.11 (1.09, 1.13)	<0.001
**Site of Service**	Office	Ref	Ref	Ref	Ref
	Outpatient Hospital	0.96 (0.94, 0.97)	<0.001	0.96 (0.95, 0.97)	<0.001
	Other	0.97 (0.93, 1.01)	0.098	1.24 (1.21, 1.27)	<0.001
**High-Deductible Health Plan**	No	Ref	Ref	Ref	Ref
	Yes	1.57 (1.54, 1.59)	<0.001	1.20 (1.19, 1.22)	<0.001
	Gastrointestinal	Ref	Ref	Ref	Ref

**Primary Disease Category**	Hematologic	0.51 (0.48, 0.54)	<0.001	0.98 (0.94, 1.02)	0.281
	Oncologic	0.43 (0.41, 0.45)	<0.001	0.94 (0.92, 0.97)	<0.001
	Ophthalmologic	1.15 (1.09, 1.21)	<0.001	0.06 (0.06, 0.07)	<0.001
	Rheumatologic	0.85 (0.82, 0.87)	<0.001	1.07 (1.06, 1.09)	<0.001
	Renal	0.40 (0.36, 0.45)	<0.001	2.57 (2.37, 2.80)	<0.001
	Other	0.55 (0.53, 0.58)	<0.001	0.87 (0.85, 0.90)	<0.001

B. OOP spending per claim

		All drugs			
		Model of Nonzero OOP Costs (N = 586,493)		Model of Mean Nonzero OOP Costs (N = 149,701)	
		Odds Ratio (95% CI)	p-value	Mean Ratio (95% CI)	p-value
**Drug Type**	Reference Biologic	Ref	Ref	Ref	Ref
	Biosimilar	1.14 (1.11, 1.16)	<0.001	0.92 (0.91, 0.94)	<0.001
**US Census Region**	Northeast	Ref	Ref	Ref	Ref
	Midwest	1.23 (1.20, 1.26)	<0.001	1.06 (1.04, 1.08)	<0.001
	South	1.30 (1.26, 1.33)	<0.001	1.17 (1.15, 1.20)	<0.001
	West	1.39 (1.35, 1.43)	<0.001	1.04 (1.02, 1.06)	<0.001
**Site of Service**	Office	Ref	Ref	Ref	Ref
	Outpatient Hospital	0.51 (0.50, 0.52)	<0.001	1.06 (1.04, 1.08)	<0.001
	Other	0.71 (0.69, 0.72)	<0.001	1.17 (1.15, 1.20)	<0.001
**High-Deductible Health Plan**	No	Ref	Ref	Ref	Ref
	Yes	1.06 (1.05, 1.08)	<0.001	1.17 (1.16, 1.18)	<0.001
**Primary Disease Category**	Gastro-intestinal	Ref	Ref	Ref	Ref
	Hematologic	0.46 (0.43, 0.49)	<0.001	1.17 (1.16, 1.18)	<0.001
	Oncologic	0.32 (0.30, 0.34)	<0.001	1.17 (1.16, 1.18)	<0.001
	Ophthalmo-logic	4.99 (4.65, 5.37)	<0.001	1.17 (1.16, 1.18)	<0.001
	Rheumato-logic	0.90 (0.88, 0.91)	<0.001	1.04 (1.02, 1.05)	<0.001
	Renal	0.72 (0.65, 0.80)	<0.001	0.56 (0.52, 0.61)	<0.001
	Other	0.65 (0.62, 0.68)	<0.001	0.91 (0.89, 0.94)	<0.001
**Month**	January	14.69 (14.21, 15.19)	<0.001	2.00 (1.95, 2.04)	<0.001
	February	6.97 (6.74, 7.20)	<0.001	1.56 (1.52, 1.60)	<0.001
	March	4.01	<0.001	1.10	<0.001

		(3.88, 4.14)		(1.07, 1.13)	
	April	2.93<br>(2.84, 3.03)	<0.001	1.02<br>(0.99, 1.05)	0.124
	May	2.20<br>(2.13, 2.28)	<0.001	0.95<br>(0.93, 0.98)	<0.001
	June	1.87<br>(1.81, 1.94)	<0.001	0.94<br>(0.92, 0.97)	<0.001
	July	1.62<br>(1.57, 1.68)	<0.001	0.99<br>(0.96, 1.02)	0.417
	August	1.37<br>(1.32, 1.42)	<0.001	0.93<br>(0.91, 0.96)	<0.001
	September	1.22<br>(1.18, 1.26)	<0.001	0.97<br>(0.94, 1.00)	0.033
	October	1.13<br>(1.09, 1.17)	<0.001	0.95<br>(0.92, 0.98)	<0.001
	November	0.99<br>(0.96, 1.03)	0.593	0.96<br>(0.93, 0.98)	0.001
	December	Ref	Ref	Ref	Ref
**Year**	2013	Ref	Ref	Ref	Ref
	2014	0.65<br>(0.57, 0.74)	<0.001	1.27<br>(1.13, 1.42)	<0.001
	2015	0.61<br>(0.53, 0.69)	<0.001	1.25<br>(1.11, 1.40)	<0.001
	2016	0.50<br>(0.44, 0.57)	<0.001	1.43<br>(1.28, 1.61)	<0.001
	2017	0.47<br>(0.42, 0.53)	<0.001	1.35<br>(1.21, 1.51)	<0.001
	2018	0.50<br>(0.45, 0.57)	<0.001	1.40<br>(1.26, 1.57)	<0.001
	2019	0.56<br>(0.50, 0.64)	<0.001	1.33<br>(1.19, 1.48)	<0.001
	2020	0.59<br>(0.52, 0.66)	<0.001	1.23<br>(1.10, 1.37)	<0.001
	2021	0.66<br>(0.58, 0.75)	<0.001	1.14<br>(1.02, 1.28)	0.019
