# Supplementary material for: Patient Out-of-Pocket Costs for Biologic Drugs After Biosimilar Competition
Source: JAMA Health Forum. 2024 Mar 29;5(3):e235429. doi: 10.1001/jamahealthforum.2023.5429 (PMC10980968; doi:10.1001/jamahealthforum.2023.5429)
Supplement: Supplement 2. — Data Sharing Statement [file jamahealthforum-e235429-s002.pdf]

Data Sharing Statement

Feng. Patient Out-Of-Pocket Costs for Biologic Drugs After Biosimilar Competition. *JAMA Health Forum*. Published March 15, 2024. doi:10.1001/jamahealthforum.2023.5429

Data
Data available: No

Additional Information
Explanation for why data not available: In line with HIPAA regulations, our data use agreements for Optum do not permit us to share patient-level source data or data derivatives with individuals and institutions not covered under the agreements. The administrative and clinical research databases used in this study are accessible to other researchers by contacting the data providers and acquiring data use agreements/licenses. The research data and data derivatives cannot be shared outside of the terms of these agreements. The data providers we used are responsive to requests for contracting use of their patient data resources. However, the cost, timeframe, and process for completing the contract for authorized use of these data varies. Contacts and information on how to acquire access to source data: Optum Clinformatics connected@optum.com
https://www.optum.com/business/solutions/life-sciences/real-world-data/claims-data.html
